# Knocking out p38α+p38β+p38γ is required to abort the myogenic program in C2C12 myoblasts and to impose uncontrolled proliferation

**DOI:** 10.1016/j.jbc.2025.108281

**Published:** 2025-02-06

**Authors:** Navit Mooshayef, Nechama Gilad, Manju P. Mohanam, David Engelberg

**Affiliations:** 1 Department of Biological Chemistry, Alexander Silberman Institute of Life Sciences, The Hebrew University of Jerusalem, Jerusalem, Israel; 2 CREATE-NUS-HUJ Mechanisms of Liver Inflammatory Diseases, National University of Singapore, 1 CREATE WAY, Innovation Wing, Singapore; 3 Department of Microbiology, Yong Loo Lin School of Medicine, National University of Singapore, Singapore

**Keywords:** MAPKs, p38alpha, p38beta, p38gamma, myoblast-to-myotubes differentiation, myogenesis, cell cycle arrest, C2C12

## Abstract

The p38 MAPKs’ family includes four isoforms, of which only p38α has been considered essential for numerous important processes including mice embryogenesis. It is also considered essential for myoblast to myotube differentiation, as exposure of myoblasts to p38**α/β** inhibitors or to siRNA that targets p38α suppresses the process. The functions of p38**β** and p38**γ** in myoblast differentiation are not clear. We knocked out p38α in C2C12 myoblasts, assuming that the resulting C2p38α^−/−^ cells would not differentiate. They did, however, form mature fibers. We found elevated levels and activation of the p38 activator MKK6 in the C2p38α^−/−^ cells, leading to activation of p38**β** and p38**γ**, which are not active in differentiating parental C2C12 cells. Thus, p38α is an inhibitor of p38**β**+p38**γ**, which perhaps replace it in promoting differentiation. To test this notion, we generated C2p38**α/γ**^−/−^ and C2p38**α/β**^−/−^ cells and found that in both clones, the myogenic program was induced. C2p38**β/γ**^−/−^ cells also formed myotubes. These observations could be interpreted in two ways: either each p38 isoform can promote, by itself, the myogenic program, or p38 activity is not required at all for the process. Generating C2p38**α/β/γ**^−/−^ cells in which the myogenic program was shut-off altogether, showed that p38 activity is critical for differentiation. Notably, C2p38**α/β/γ**^−/−^ cells proliferate uncontrollably and give rise to foci, reminiscence of oncogenically transformed cells. In summary, our study shows that a crosstalk between p38 isoforms functions in C2C12 cells as a safeguard mechanism that ensures resilience of the p38 activity in promoting the myogenic program and enforcing cell cycle arrest.

Myoblast to myotube differentiation is the final step in the formation of functional muscles in the developing embryo. This process involves arrest of cell division, and fusion of myoblasts into elongated multinucleated cells, termed myotubes. The process is controlled primarily by four transcriptional activators, MyoD, Myf5, myogenin, and MRF4, collectively known as myogenic regulatory factors (MRFs) (1, 2, 3). They are sequentially activated and in turn induce expression of proteins that form the myogenic contractile apparatus, such as myosin heavy chain (MyHC), myosin light chains, and α-skeletal actin, proteins associated with the myogenic energy metabolism, such as creatine kinase (1, 2, 3), and proteins required for myoblast fusion to multinuclear fibers, for example, myomerger and myomaker (4, 5, 6, 7). MRFs are also involved in inducing the withdrawal from cell cycle and growth arrest, which is a pre-requisite for differentiation (8, 9). This is obtained by downregulation of cyclin D1 expression, a cyclin that promotes progress from the G0/G1 to the S phase of the cell cycle (10, 11).

Myoblast to myotube differentiation, including activation of the proper myogenic program, could be induced in myogenic cell lines such as C2C12 (12, 13) and L8 (14), by providing the cells with low serum, a medium known as differentiation medium (DM). Several signaling pathways are induced by DM and evoke the myogenic program (15). The MAP kinase p38α signaling cascade was reported to be critical for this process (11, 16, 17, 18). Two more isoforms of the p38 family, p38β and p38γ, are also expressed in skeletal muscle and in myogenic cell lines (19, 20, 21), but their role in myoblast to myotube differentiation is not clear. In fact, relatively little is known in general about the roles of these isoforms, as well as of the fourth isoform, p38δ. p38α is ubiquitously expressed, is functional in most tissues, and is critical for various physiological processes including embryonic development (22, 23, 24). p38γ and p38δ on the other hand, are expressed primarily in striated muscle and endocrine glands, respectively, and are not essential for the functionality of these or other tissues. Mice knocked out for p38γ or p38δ, or even for p38γ+p38δ, are viable and fertile (19, 25). Very little is known about the role of p38β, which is expressed at low levels in most tissues, with some higher levels in the brain (22). Although highly similar to p38α, p38β may have specific roles, as p38α/β^−/−^ mice manifest phenotypes that are not observed in each knockout alone (26). Also, p38β can rescue just some of the defects of p38α^−/−^ mice, even when expressed from the p38α promoter. For example, it cannot rescue embryonic death of these mice (26). In any case, the putative specific roles of p38β are clearly not essential, as p38β^−/−^ mice are viable, fertile, and show no particular abnormalities (27).

p38α was demonstrated to be important not only for myoblast to myotube differentiation but also for other processes in skeletal muscle, such as development (11, 28, 29, 30). It is also associated with aging of muscle stem cells (MuSCs) (31, 32), although its exact role in muscle aging is yet to be revealed (33). In the myogenic cell lines C2C12 and L8, several observations suggested that p38α is essential for myoblast to myotube differentiation. Its activity was reportedly elevated in response to differentiation signals (17, 34), and exposure of the cells to p38α/β-specific pharmacological inhibitors was reported to abolish differentiation (11, 17, 34, 35, 36, 37). Knocking down p38α in C2C12 cells using siRNA also impaired differentiation (16, 37, 38). Accordingly, primary cultures of myoblasts originating from mice lacking p38α did not differentiate to multinucleated myofibers (11, 39), and primary cultures prepared from WT mice failed to differentiate in the presence of the p38α/β inhibitor SB203580 (11).

Intriguingly, a single report showed that knocking down p38β or p38γ individually in C2C12 cells by siRNA significantly reduced differentiation, suggesting that each is required for proper differentiation (16). However, mice knocked out for either p38β, p38γ, or p38δ are healthy and fertile, developing functional muscle tissues (19, 27). Also, primary cultures of myoblasts prepared from mice lacking p38β, p38γ, or p38δ did differentiate, although differentiation of p38γ^−/−^ cells was not as efficient as that of WT cells (11). Finally, mice knocked out for p38γ developed fewer fibers following damage and contained a lower number of MuSC, suggesting that p38γ is responsible for transient proliferation of MuSC in response to damage, probably by direct phosphorylation and inhibition of MyoD (40). These studies combined point at p38α as the only p38 isoform critical for myoblast to myotube differentiation *in vitro* and *in vivo*.

Despite the many observations that assign to p38α an essential role in myoblasts differentiation, some studies suggested that it may not be essential for the process. It seems, for example, that at late stages of differentiation, p38α/β is in fact a suppressor rather than a promoter of differentiation, as it inhibits transcription of myogenic-related genes by phosphorylating MRF4 (41). In addition, primary limb mesenchymal cultures treated with p38 inhibitor showed an increase in myogenic markers and myotube formation (41, 42). More astonishing, mice in which p38α was conditionally knocked out in muscle at an early embryonic stage developed intact muscle (28). Indeed, some defects, such as low muscle mass and abnormally large populations of MuSCs, were observed in these mice 3 weeks after birth, but these phenotypes seem to be related to overactivation of p38γ in the p38α KO muscle (28). Notably, p38γ is phosphorylated following p38α knockout or inactivation (28, 43), but the role of this phosphorylation is not defined.

Seeking less ambiguous understanding of the role of p38 isoforms in myoblast to myotube differentiation, we systematically knocked out p38 isoforms in C2C12 cells individually and in combination. Given the reports that p38α/β inhibitors or siRNAs which specifically target p38α abolish differentiation, the presumption was that knocking out p38α would abolish differentiation altogether. In contrast to this premise, the myogenic program in C2p38α^−/−^ was almost unaffected and cells developed mature myotubes. C2p38β^−/−^ and C2p38γ^−/−^ cell lines also differentiated efficiently. We show that p38α^−/−^ myoblasts are capable of differentiation because p38β and p38γ, which are not active in parental C2C12 cells, are phosphorylated/activated in them as a result of elevated MKK6 levels. In fact, p38β or p38γ alone are capable of activating the myogenic program as doubly knocked out p38α/β^−/−^ and p38α/γ^−/−^ cells execute the myogenic program. These results point at suppression of p38β and p38γ by p38α, and desuppression when p38α is inactive. Only when the three isoforms—p38α, p38β, and p38γ—were knocked out together the myogenic program was not induced. This seems to occur because C2p38αβγ^−/−^ cells cannot withdraw from the cell cycle. They enter a mode of uncontrolled proliferation and form foci reminiscence of oncogenically transformed cells. These results together suggest that p38 isoforms can compensate for the activity of each other in promoting myoblast to myotube differentiation and that an important role of p38 in the process is supporting cell cycle withdrawal.

## Results

### C2C12 cells lacking p38**α** differentiate to mature myotubes

**Figure 1:**
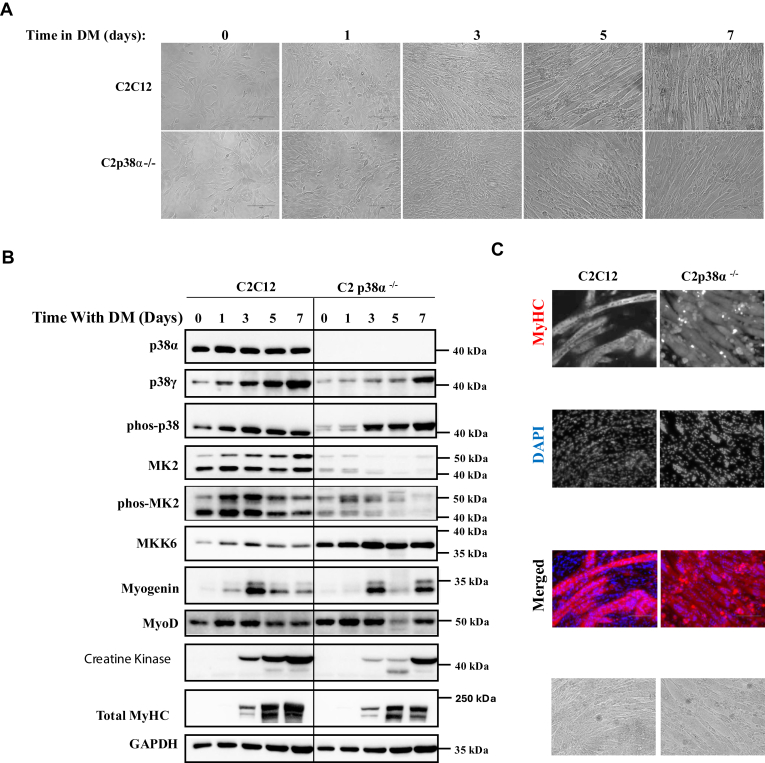
**C2p38α**^**−/−**^**cells are capable of executing myoblast to myotube differentiation and of expression of myogenic markers, perhaps through phosphorylation of other p38 isoforms.** C2C12 and C2p38α^−/−^ cells were cultured in growth medium (GM) to subconfluency. The medium was then changed to differentiation medium (DM) for additional 7 days. *A*, cells were photographed at the indicated days. *B*, protein lysates were prepared at the indicated time points. Twenty micrograms of protein lysates were separated by SDS-PAGE, blotted, and probed with the indicated antibodies. *C*, cells were fixed at day 7, immune-stained with anti MyHC antibody, and also stained with DAPI. DAPI, 4′,6-diamidino-2-phenylindole.

As inhibition or downregulation of p38α in C2C12 myoblasts was reported to abolish their ability to differentiate (11, 17, 34, 35, 36, 37, 39), it was assumed that knocking out p38α in these cells would have the same effect. p38α was thus knocked out using the CRISPR-Cas method, and the resulting cells were termed as C2p38α^−/−^ cells (for details see “Experimental procedures” and Fig. S1). In contrast to the premise, when exposed to DM, C2p38α^−/−^ cells differentiated efficiently and formed mature multinucleated myotubes, although their differentiation was somewhat delayed compared to that of parental C2C12 cells (Fig. 1*A*). Accordingly, expression of the MRFs MyoD and myogenin, as well as of proteins required for muscle function such as creatine kinase and MyHC, was induced during the differentiation of C2p38α^−/−^ cells to levels similar to those in parental C2C12 cells (Fig. 1, *B* and *C*). On the other hand, expression of MK2, a downstream target of p38α, was downregulated in C2p38α^−/−^ cells as expected (Fig. 1*B*). Immunofluorescence staining with anti-MyHC antibodies and nuclei staining with 4′,6-diamidino-2-phenylindole (DAPI) showed that both parental C2C12 and C2p38α^−/−^ cells developed multinucleated myotubes that expressed MyHC at similar levels (Fig. 1*C*). Thus, it seems that the myogenic program in C2C12 cells is fully operational in the complete absence of p38α.

### MKK6 activates other p38 isoforms in the course of the differentiation of C2p38**α**^−/−^ cells

The fact that C2p38α^−/−^ cells differentiate in a manner similar to parental C2C12 cells may suggest that p38 activity is not required for the process. However, probing protein lysates prepared from C2p38α^−/−^ cells with anti-phospho(TGY)-p38 antibody revealed that *de novo* p38 activity (*i.e.*, activity that is not observed in parental C2C12 cells) exists in C2p38α^−/−^ cells. The anti-phospho(TGY)-p38 antibodies gave rise to two bands on the Western blot that migrated slower than p38α (Fig. 1*B*, third row; for quantification of the phosphorylation and expression levels, see Fig. S2, *C*, *D* and *F*). This observation raises the possibility that other p38 isoforms, most probably p38β and p38γ, compensate for the missing p38α in promoting differentiation. Phosphorylation of these molecules is a result of upregulation and activation (phosphorylation) of the p38 upstream kinase MKK6 in C2p38α^−/−^ cells (Fig. 1*B*, fifth row; Fig. S2, *A*, *B*, *D* and *E*; for quantification, see Fig. S2*D*). The combination of its elevated level and activity with the absence of p38α, makes MKK6 more available to other p38 isoforms, its sole substrates in C2p38α^−/−^ cells.

We checked, therefore, whether p38β, p38γ, or perhaps both together, indeed contribute to the myoblast to myotube differentiation of C2p38α^−/−^ cells.

### C2C12 cells knocked out for p38**γ**, or for both p38**α** and p38**γ** still differentiate

**Figure 2:**
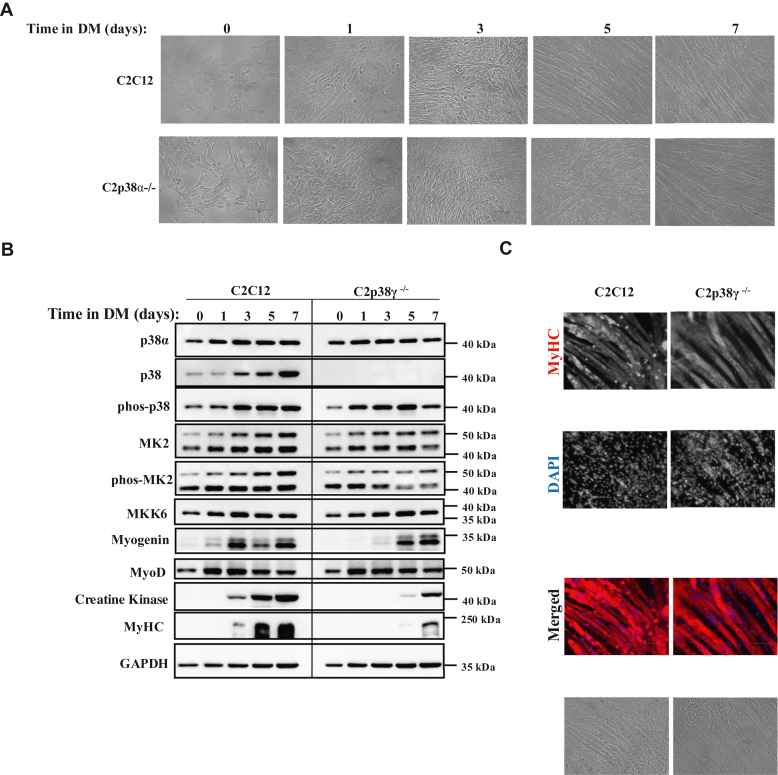

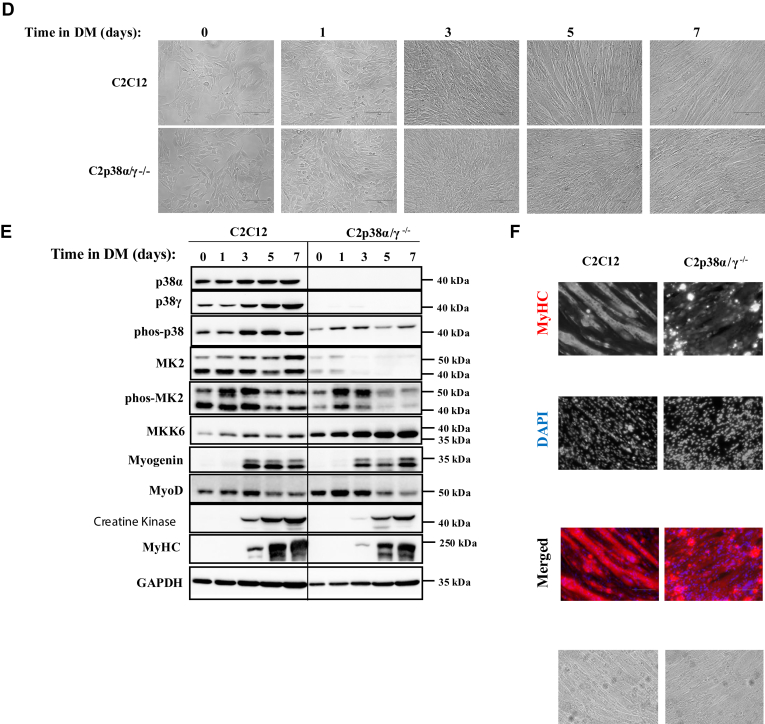
**C2p38γ**^**−/−**^**and C2p38α/γ**^**−/−**^**cells are capable of executing myoblast to myotube differentiation and of expression of myogenic markers.***A–C*, C2C12 and C2p38γ^−/−^ cells were cultured in growth medium (GM) to subconfluency. The medium was then changed to differentiation medium (DM) for additional 7 days. *A,* cells were photographed at the indicated days. *B*, protein lysates were prepared at the indicated time points. Twenty micrograms of protein lysates were separated by SDS-PAGE, blotted and probed with the indicated antibodies. *C*, cells were fixed at day 7 and immune-stained with anti MyHC antibody, and also stained with DAPI. *D–F*, C2C12 and C2p38α/γ^−/−^ cells were cultured in GM to subconfluency. The medium was then changed to DM for additional 7 days. *D*, cells were photographed at the indicated days. *E*, protein lysates were prepared on the indicated days. Twenty micrograms of protein of each cell lysate were separated by SDS-PAGE, blotted, and probed with the indicated antibodies. *F*, cells were fixed at day 7 and immune-stained with anti MyHC antibody and also stained with DAPI. DAPI, 4′,6-diamidino-2-phenylindole; MyHC, myosin heavy chain.

p38γ is expressed at high levels in myogenic cells (44) as well as in C2p38α^−/−^ cells (Fig. 1*B*, second row), making it the prime candidate as an activated isoform in C2p38α^−/−^ cells. To check the possibility that p38γ plays a role in the differentiation of C2p38α^−/−^ cells, and perhaps also of C2C12 cells, we knocked out the gene encoding p38γ in the two cell lines (Figs. S3 and S4) and monitored the myogenic markers in the resulting C2p38γ^−/−^ and C2p38α/γ^−/−^ cells. When exposed to DM, C2p38γ^−/−^ cells showed lower expression of myogenin, CK, and MyHC (Fig. 2*B*), but did differentiate to multinucleated myotubes (Fig. 2, *A* and *C*). Thus, elimination of p38γ from C2C12 cells has little effect on their differentiation capabilities. The double KO C2p38α/γ^−/−^ cells were found to have a somewhat different appearance compared to parental C2C12 cells, manifesting a more spherical morphology (Fig. 2*D*), but were nevertheless clearly capable of differentiation to thick multinuclear myofibers (Fig. 2, *E* and *F*). Also, MyoD, MyHC, and CK were expressed during differentiation of C2p38α/γ^−/−^ cells at almost similar levels and kinetics as in parental C2C12 cells (Fig. 2*E*). Myogenin levels seemed to be a bit lower (Fig. 2*E*).

These observations show that although activated in C2p38α^−/−^ cells, p38γ is dispensable for their differentiation. It could be that p38 activity is not required for differentiation of C2p38α^−/−^ cells, or that yet another isoform, perhaps p38β, could support differentiation. Indeed, lysates prepared from C2p38α/γ^−/−^ cells still reacted weakly with anti-phospho(TGY)-p38 antibody and also showed high levels of MKK6 that explains this phosphorylation (Fig. 2*E*; for quantification, see Fig. S2, *C* and *F*).

### p38**α/β** inhibitors impair differentiation of C2p38**α**^−/−^ cells

**Figure 3:**
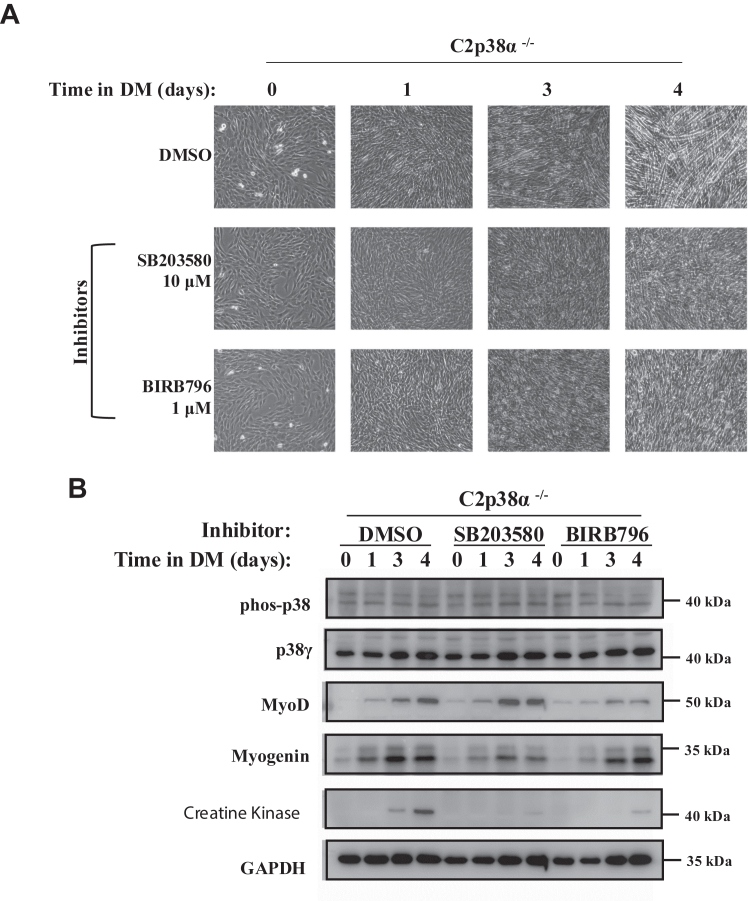
**A pan-p38 inhibitor, as well as a p38α/β-specific inhibitor, impairs differentiation of C2p38α**^**−/−**^**cells.** C2p38α^−/−^ cells were cultured in growth medium to subconfluency. The medium was then changed to differentiation medium (DM) for additional 5 days. The DM was supplemented with either DMSO, 1 μM of BIRB796, or 10 μM of SB203580. *A*, cells were photographed at the indicated days following beginning of treatment. *B*, lysates were prepared at the indicated time points. Twenty micrograms of protein lysates were separated by SDS-PAGE, blotted, and probed with the indicated antibodies. DMSO, dimethyl sulfoxide.

To obtain an indication as to whether p38 activity is at all required for differentiation of C2p38α^−/−^ cells, we exposed these cells to the pan-p38 inhibitor BIRB796. To test whether p38β is specifically required for differentiation of C2p38α^−/−^ cells, we exposed them to the p38α/β-specific inhibitor SB203580. Microscopic inspection showed that after 4 days of treatment, cells not exposed to inhibitors formed myotubes (Fig. 3*A*, upper row), while cells exposed to either BIRB796 or SB203580 did not (Fig. 3*A*). At the biochemical level the myogenic program was induced to some extent in the presence of the inhibitors. Expression of MyoD and myogenin was quite high, but that of creatine kinase was barely detectable (Fig. 3*B*). Thus, C2p38α^−/−^ cells cannot induce myogenesis properly and form myotubes without p38 activity. Inhibition of differentiation with SB203580 strengthens the notion that p38β activity is important for C2p38α^−/−^ differentiation because it is the major existing target for SB203580 in these cells (SB203580 is significantly less efficient toward p38γ (45, 46)).

The possibility nevertheless remains that SB203580 inhibits, nonspecifically, some other unknown targets and blocks differentiation *via* this mechanism. Therefore, to unequivocally address the role of p38β, we established C2p38β^−/−^ and C2p38αβ^−/−^ cells (Figs. S5 and S6) and tested their differentiation capabilities.

### C2C12 cells knocked out for both p38**α** and p38**β** induce expression of myogenic components, but do not form myotubes

**Figure 4:**
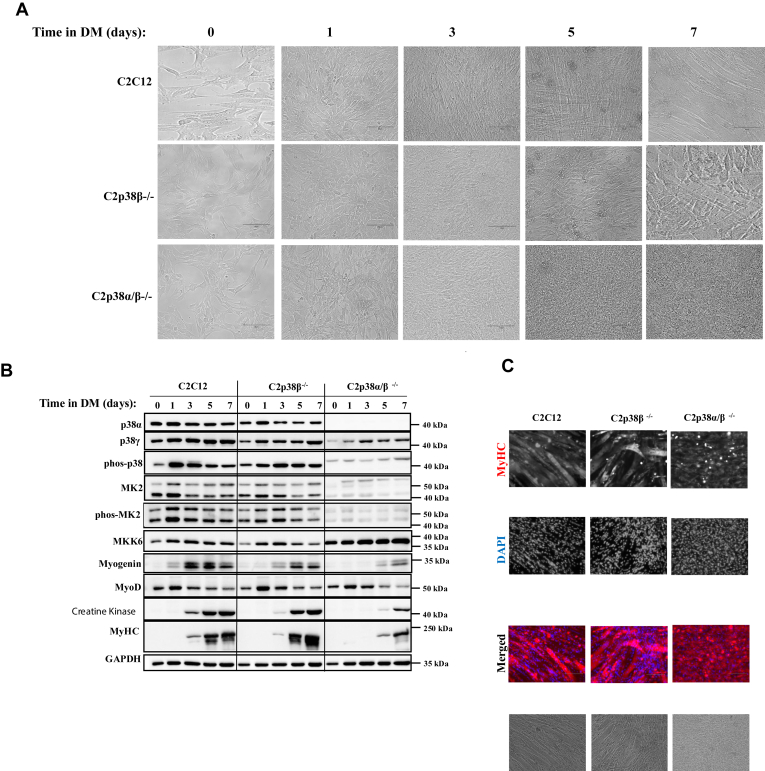
**C2p38β**^**−/−**^**cells are capable of executing a complete myoblast to myotube differentiation, but C2p38α/β**^**−/−**^**cells are not.** C2C12, C2p38β^−/−^, or C2p38α/β^−/−^ cells were cultured in growth medium to subconfluency. The medium was then changed to differentiation medium (DM) for additional 7 days. *A*, cells were photographed at the indicated days. *B*, protein lysates were prepared at the indicated time points. Twenty micrograms of protein lysates were separated by SDS-PAGE, blotted, and probed with the indicated antibodies. *C*, cells were fixed at day 7 and immune-stained with anti MyHC antibody and also stained with DAPI. DAPI, 4′,6-diamidino-2-phenylindole; MyHC, myosin heavy chain.

When exposed to DM C2p38β^−/−^ cells differentiated normally and formed mature fibers (Fig. 4, *A* and *C*). They expressed the relevant proteins at some reduced levels compared to those in C2C12 cells that were tested in parallel (Fig. 4*B*, middle panel). The doubly knocked out C2p38αβ^−/−^ cells also expressed myogenin, MyoD, MyHC, and creatine kinase (Fig. 4*B*, right panel). Although levels of these proteins were lower than parental C2C12 and C2p38β^−/−^ cells, they were readily detectable, suggesting that the myogenic program is functional. Accordingly, immunostaining with anti-MyHC antibodies resulted in a strong signal (Fig. 4*C*, right panel). However, multinucleated fibers were not observed *via* microscopic inspection of the C2p38αβ^−/−^ (Fig. 4, *A*, lower panel and *C* right panel).

It is concluded that p38γ can, by itself, induce the myogenic program, except for the fusion step. As C2p38α^−/−^ and C2p38αγ^−/−^ cells do form myotubes, p38β is a major factor in supporting the completion of differentiation, including the fusion step, in p38α^−/−^ myoblasts.

### p38α *per se* is sufficient to promote myoblast to myotube differentiation in C2C12 cells

**Figure 5:**
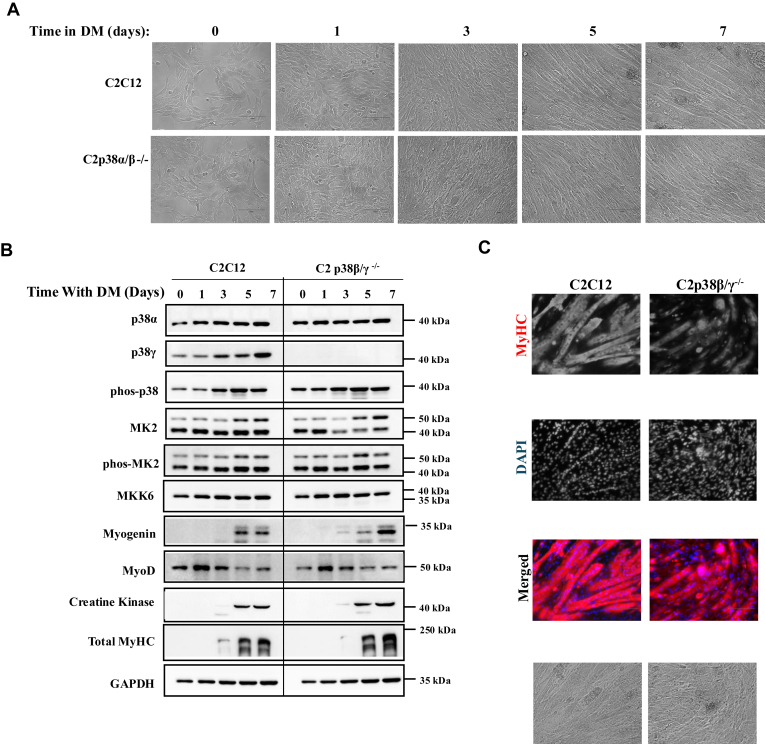
**C2p38β/γ**^**−/−**^**cells are capable of executing myoblast to myotube differentiation and of expression of myogenic markers.** C2C12 and C2p38β/γ^−/−^ cells were cultured in growth medium (GM) to subconfluency. The medium was then changed to differentiation medium (DM) for additional 7 days. *A*, cells were photographed at the indicated days. *B*, protein lysates were prepared at the indicated time points. Twenty micrograms of protein lysates were separated by SDS-PAGE, blotted, and probed with the indicated antibodies. *C*, cells were fixed at day 7 and immune-stained with anti MyHC antibody and also stained with DAPI. DAPI, 4′,6-diamidino-2-phenylindole; MyHC, myosin heavy chain.

The observations that important parts of the myogenic program are induced in C2p38α/β^−/−^ and C2α/γ^−/−^ cells show that p38β or p38γ can each activate the differentiation machinery by itself. To test whether p38α could also promote differentiation by itself, we created C2p38β/γ^−/−^ cells, as described in Fig. S7. These C2p38β/γ^−/−^ cells differentiated normally to multinuclear myotubes that also expressed MyHC (Fig. 5, *A*–*C*). Of note, the expression levels of MyoD were slightly lower and the level of phosphorylated p38α is slightly higher in the C2p38β/γ^−/−^ cells than those in parental C2C12 cells. The expression of MyHC is similar in C2C12 and C2p38β/γ^−/−^ cells. Thus, just like p38β and p38γ, p38α *per se* can support activation of the myogenic program.

### C2C12 cells knocked out for p38**α**+p38**β**+p38**γ** do not differentiate

The observations that C2C12 cells that lack two p38 isoforms still induce the myogenic program may suggest that each p38 isoform expressed in muscle can promote by itself activation of the myogenic program. Another explanation for the observation is that p38 activity is not required whatsoever.

**Figure 6:**
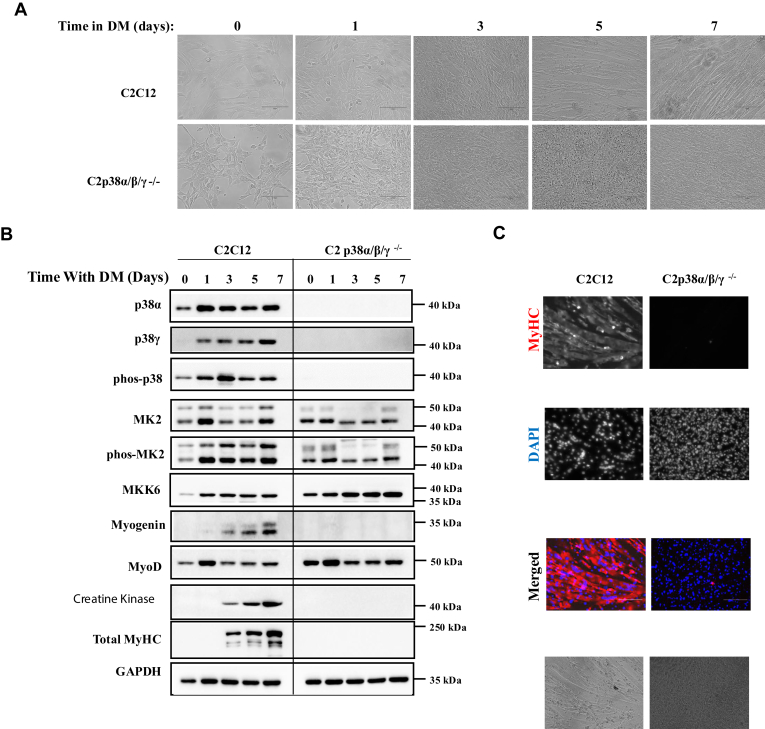
**C2p38α/γ/β**^**−/−**^**cells do not undergo myoblast to myotube differentiation and do not induce expression of myogenic proteins.** C2C12 and C2p38α/γ/β^−/−^ cells were cultured in growth medium (GM) to subconfluency. The medium was then changed to differentiation medium (DM) for additional 7 days. *A*, cells were photographed at the indicated days. *B*, protein lysates were prepared at the indicated time points. Twenty micrograms of protein lysates were separated by SDS-PAGE, blotted, and probed with the indicated antibodies. *C*, cells were fixed at day 7 and immune-stained with anti MyHC antibody and also stained with DAPI. DAPI, 4′,6-diamidino-2-phenylindole; MyHC, myosin heavy chain.

To challenge unambiguously the essentiality of p38 activity for differentiation, we generated C2C12 cells lacking the three p38 isoforms α, β, and γ (Fig. S8). No phosphorylation of p38 was detected in the triply knocked out C2p38α/β/γ^−/−^ cells (Fig. 6*B*), confirming no p38 activity. When exposed to DM C2p38α/β/γ^−/−^ cells failed to exhibit any sign of multinucleated myotubes even after 7 days (Fig. 6, *A* and *C*). Immunostaining and western blot analysis revealed almost no expression of MyHC, creatine kinase, and myogenin (Fig. 6, *B* and *C*). Of the myogenic components tested, only MyoD was expressed, at levels even higher than those in parental C2C12 cells (Fig. 6*B*). It is concluded that p38 activity is critical for the execution of the myogenic program.

Properties of all knockout derivatives of C2C12 cells are summarized in Table 1.

**Table 1:** Properties of parental and knocked out derivatives of C2C12 cells

Cells	Multinuclear myotubes	MyoD	Myogenin	CK	MyHC	Myomerger	Myomaker	Cyclin D1
C2C12	+	+	+	+	+	+	+	-
C2p38α^−/−^	+	+	+	+	+	+	+	−/+
C2p38γ^−/−^	+	+	+	−/+	−/+	+	+	−/+
C2p38α/γ^−/−^	+	+	+	+	+	+	+	+
C2p38β^−/−^	+	+	+	+	+	+	+	−/+
C2p38α/β^−/−^	-	+	+	−/+	−/+	-	-	++
C2p38β/γ^−/−^	+	+	+	+	+	+	+	−/+
C2p38α/β/γ^−/−^	-	+	-	-	-	-	-	+++

MyHC, myosin heavy chain.

### Rapid uncontrolled proliferation of C2p38**α/β/γ**^−/−^ cells leads to appearance of foci

**Figure 7:**
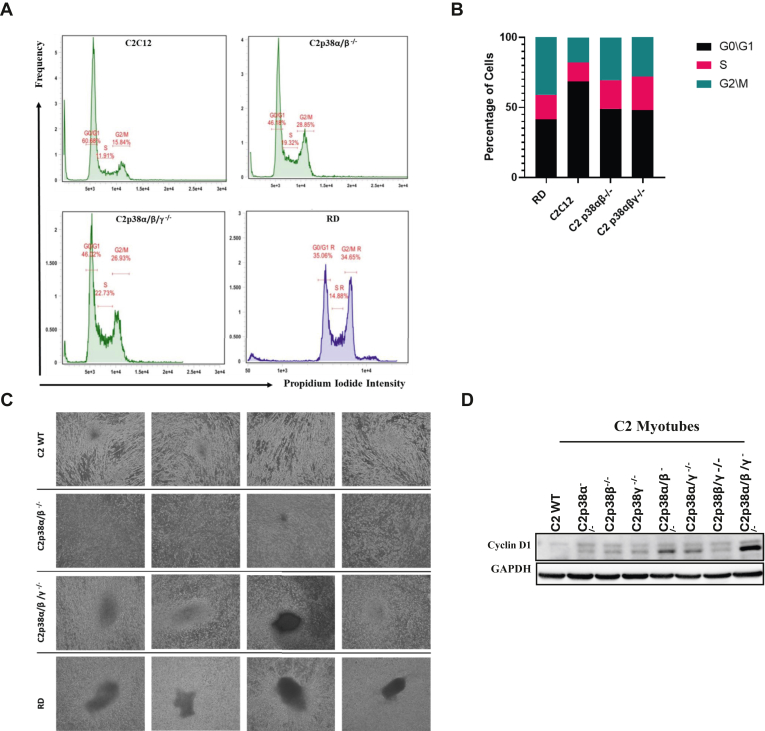
**Higher rate of C2p38α/γ/β**^**−/−**^**cells reside in S phase of the cell cycle, express higher levels of cyclin D1 and give rise to foci.***A*, cell cycle analysis of the indicated cells. Cells were stained with propidium iodide (PI) and then single cells were analyzed for PI intensity. The frequency of each population in the different cell cycle phases was monitored. *B*, percentage of cells in each cell cycle phase was calculated for the cells described in (**A**). *C*, C2C12, C2p38α/β^−/−^, C2p38α/β/γ^−/−^, and RD cells were allowed to grow for 18 days in GM. Representative images of the culture are presented including appearance of foci. *D*, cells of parental C2C12 and the indicated knocked out derivatives were incubated in DM for 5 days. Lysates were tested by western blot with the indicated antibodies. Note that the GAPDH panel is the same as in Figs 8*C* and S2*E*, as the Western blots shown in these figures were performed together on the same lysates. GM, growth medium; RD, rhabdomyosarcoma; DM, differentiation medium.

Why is the myogenic program not induced in C2p38α/β/γ^−/−^ cells? As a prerequisite for differentiation is withdrawal from cell division and as p38s are known to induce cell cycle arrest (8, 9, 11, 39, 47, 48), including during muscle differentiation (39, 47, 49), we tested the cell cycle profile of parental C2C12 and the derived knocked out clones. As a control for nondifferentiating myoblasts, we also tested a rhabdomyosarcoma (RD) cell line. FACS analysis revealed a decrease in the percent of cells residing in G0/G1 phase and an increase in cells residing in S and G2/M phases in the population of C2p38α/β^−/−^ and C2p38α/β/γ^−/−^ cells, as compared to the populations of C2C12 cells and other clones (Figs. 7, *A* and *B* for C2C12, C2p38α/β^−/−^, and C2p38α/β/γ^−/−^ cells; for FACS analysis of other clones, see Fig. S9). Furthermore, while growing C2p38α/β/γ^−/−^ cells, we noticed the appearance of tumorigenic-like foci (Fig. 7*C*). These were few in number and appeared after cultures were maintained for more than 2 weeks in the plate, but were never observed in any other clone, including C2p38α/β^−/−^, which also does not form myotubes (Fig. 7*C*). Thus, the lack of p38 causes not only inability of the cells to arrest cell cycle but also rapid uncontrolled proliferation, reminiscent of oncogenically transformed cells.

A critical cyclin that is downregulated in the course of myoblast to myotube differentiation is cyclin D1. We tested its levels in all C2C12-derived knocked out clones after 5 days in DM and found that its expression is higher in C2p38α/β^−/−^ and C2p38α/β/γ^−/−^ cells than the other clones (Fig. 7*D*). Some increase is observed in C2p38α/γ^−/−^ cells too. Yet, the level in C2p38α/β/γ^−/−^ cells was markedly higher than that in the C2p38α/β^−/−^ and C2p38α/γ^−/−^ cells, suggesting a correlation between levels of cyclin D1 expression and negative effect on activation of the myogenic program. It seems that p38α is the major regulator of cyclin D1 downregulation during differentiation, but it does not do it alone as double and triple KOs are required to cause elevation of cyclin D1 expression.

### p38**α** or p38**β**, but not p38**γ**, promote myoblast fusion by supporting expression of the fusion proteins myomerker and myomerger

**Figure 8:**
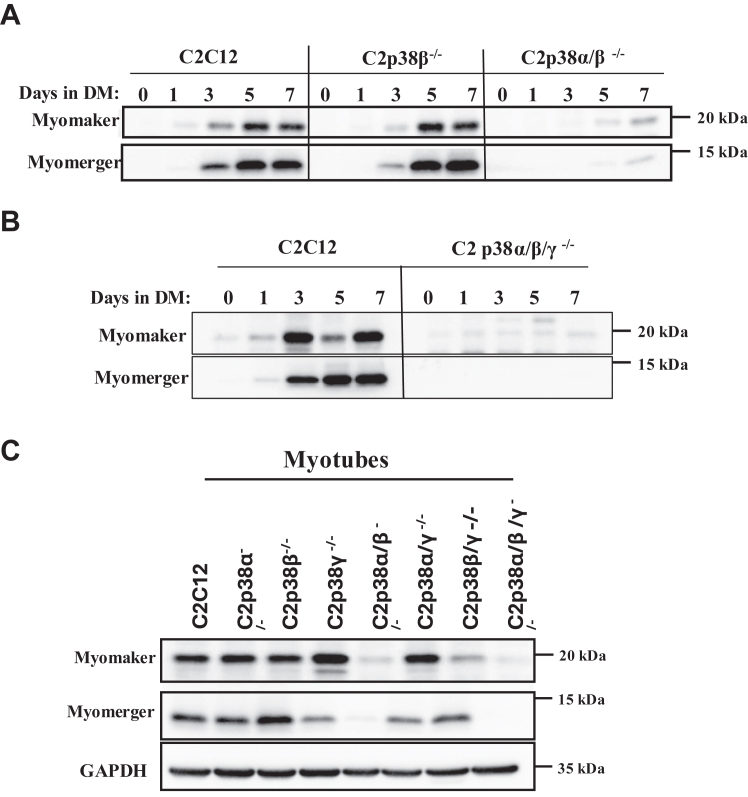
**p38α or p38β is required to induce expression of myomerger and myomaker in differentiating C2C12.** Cells were cultured in growth medium (GM) to subconfluency. The medium was then changed to differentiation medium (DM) for additional 7 days. *A* and *B*, protein lysates prepared from C2C12, C2p38β^−/−^, and C2p38α/β^−/−^ cells (A) and from C2C12 and C2p38α/β/γ^−/−^ cells (B) were tested with the indicated antibodies. *C*, parental C2C12 and the indicated C2p38KO cells were incubated in DM for 5 days. Lysates were prepared and analyzed by Western blots with the indicated antibodies. Note that the GAPDH panel is the same as in Figs 7*D* and S2*E*, as the Western blots shown in these figures were performed together on the same lysates.

The emerging conclusion from above experiments is that each of the two isoforms, p38α or p38β, is capable by itself to promote activation of all aspects of the myogenic program, while p38γ cannot support the fusion step. Myoblast fusion into myotubes is executed primarily by the proteins myomaker and myomerger, known as fusogenes (4, 5, 6, 7). These proteins are not expressed in myoblasts and emerge after 3 days in DM (Fig. 8, *A* and *B*). Monitoring the levels of myomaker and myomerger revealed that both proteins are not induced in the two clones that are incapable of fusing, C2p38α/β^−/−^ and C2p38α/β/γ^−/−^ (Fig. 8, *A* and *B*), but are induced in all other knocked out clones (Fig. 8*C*).

These results explain why p38γ, the only isoform expressed in C2p38α/β^−/−^ cells, does not support formation of myotubes, even though it can induce critical parts of the myogenic program. p38β, on the other hand, can compensate by itself for the function of p38α and induces the expression of the fusogenes.

### The p38 inhibitors SB203580 and BIRB796 cause delay, but not complete suppression of myoblast to myotube differentiation of C2C12 cells

**Figure 9:**
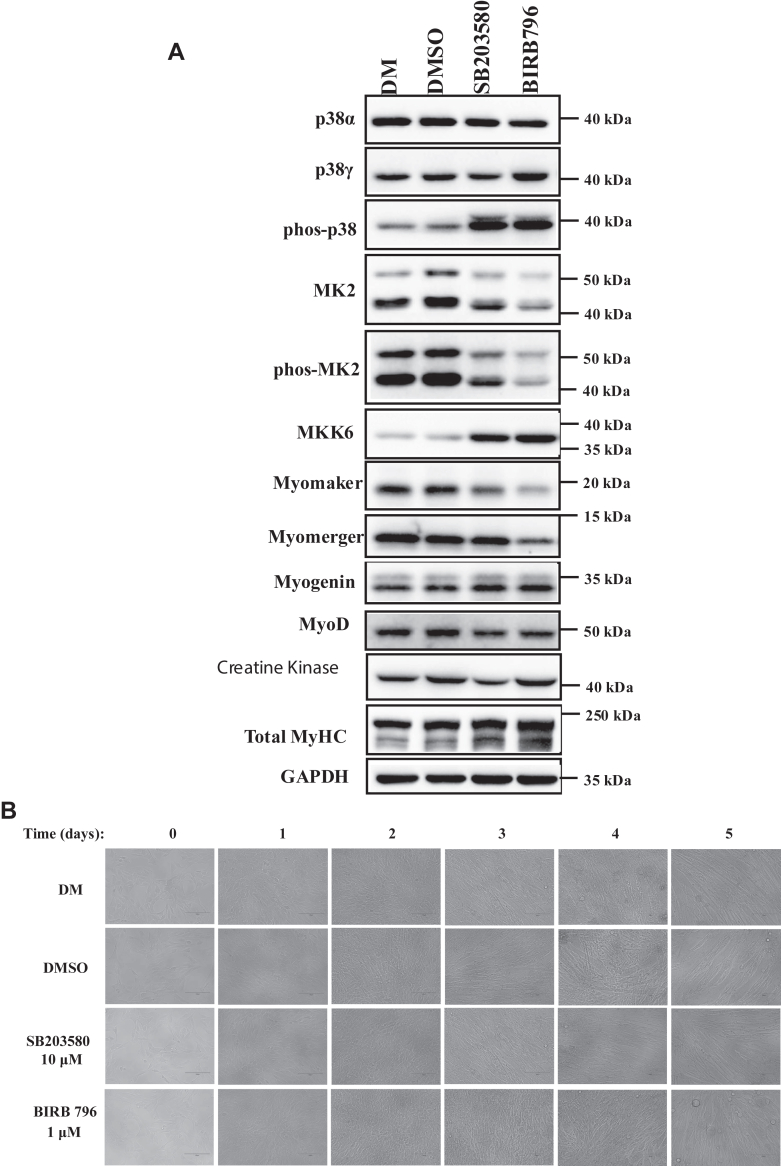
**C2C12 cells respond to p38 inhibitors by elevating levels of MKK6 and phosphorylation of p38 isoforms. Some myotubes do appear following 5 days of incubation with inhibitors.***A*, the myogenic program is functional in the presence of SB203580 and BIRB796. C2C12 cells were incubated for 5 days with either DM, DM + DMSO, or DM+ the indicated inhibitor, with addition of fresh media every 24 h. Protein lysates were prepared and analyzed by western blot with the indicated antibodies. *B*, the myoblast to myotube process of C2C12 cells is delayed and weakened, but not totally abolished. C2C12 were treated as described in (**A**) and photographed daily. DM, differentiation medium.

The observation that C2p38α^−/−^ cells form myotubes, which suggests that p38α is not essential for the process, seems at a first glance to contradict previous results that suggested that p38α is critical for differentiation. As these previous conclusions were based on the use of p38 inhibitors (17, 34, 35), we also exposed C2C12 to SB203580 and BIRB796. We noted that the previous studies exposed the cells to inhibitors for a short time (3–4 days). Perhaps this short time is not sufficient for completing a scenario similar to that observed in the C2p38α^−/−^ cells, namely elevation of MKK6 activity and in turn p38β and p38γ activation and promotion of differentiation. Therefore, in our experiment, we treated the culture with inhibitors beyond 5 days (Fig. 9). Remarkably, C2C12 cells exposed to the p38 inhibitors showed characteristics similar to those showed by C2p38α^−/−^ cells. For example, MKK6 levels were upregulated and consequently p38 phosphorylation (Fig. 9*A*). In accordance with previous reports, C2C12 cells exposed to inhibitors did not develop myotubes in the first 3 days of the experiment, while cells not provided with inhibitors did differentiate during that time (Fig. 9*B*). However, on day 5 myotubes did appear in cultures exposed to inhibitors (Fig. 9*A*; note that fresh inhibitors were added daily). Indeed, myotubes were not as developed as in cultures not exposed to inhibitors but were clearly apparent. Accordingly, myogenic markers were expressed at similar levels as in cells not exposed to inhibitors, except for myomerger and myomaker that were expressed at somewhat reduced level (Fig. 9*A*). We conclude that C2C12 cells respond to p38α knockout in a similar manner as they respond to its inhibition. The capability to induce the myogenic program and even to form small myotubes after 5 days with SB203580 can be explained by the activation of p38γ, which is less sensitive to this drug (45, 46) and perhaps also to the elevation of p38β, which is 10-fold less sensitive to SB203580 than p38α (45, 46). Formation of myotubes following 5 days in the presence of BIRB796 is more difficult to explain as this drug is considered a pan p38 inhibitor. Nevertheless, BIRB796 is a most potent p38α inhibitor, with IC50 of 7 to 50 nM (depending on the particular study), but less potent toward p38β (IC50 = 200–500 nM) and p38γ (IC50 > 300 nM) (50). Perhaps, like the case of SB203580, when the effect of activation of p38β and p38γ in response to p38α inhibition is combined with their lower sensitivity to the drug, some p38 activity still exists, sufficient to support a certain degree of differentiation.

## Discussion

The systematic KO approach presented here revealed that p38α is not essential for differentiation of C2C12 myoblasts, because a powerful backup system, involving p38β and p38γ, is aroused and replaces p38α when it becomes inactive. The observation that p38β and/or p38γ are activated only in cells in which p38α is absent (*i.e.*, C2p38α^−/−^, C2p38α/β^−/−^, and C2p38α/γ^−/−^ cells) could be interpreted as if their role in the normal myoblast to myotube differentiation process is marginal so that their major function is to serve as backups for p38α. This notion may explain the development of several p38 isoforms apart from p38α in the course of evolution. Indeed, it is hitherto not clear if p38β and p38γ execute specific functions. Although some tissue-specific activities were proposed for p38γ and p38δ, these are far from being critical. The genes encoding these isoforms can be knocked out in mice with just marginal effects on the animal’s life (19).

The riddle regarding the existence of isoforms of MAPKs extends beyond p38s. Erk1 and Erk2 seem to fulfill the very same physiological functions, and isoform-specific functions among Erks are probably minor, as each can fully compensate for the absence of the other (51, 52). In the JNK family, JNK3 might have some brain-specific functions, but JNK1 and JNK2 readily fill-in for each other (53, 54, 55).

Amongst p38 isoforms, the functions of p38β are the least defined. Unlike the situations in the Erk and JNK subfamilies, where similar isoforms replace each other, p38β cannot fully replace p38α. Although 75% identical and 88% similar to p38α (56), p38β can only partially fulfill some of p38α functions in the developing embryo (*e.g.*, lung development) (26). And yet, in C2C12 myoblasts p38β is an efficient fill-in player for p38α. Whether this kind of fill-in relationship is specific to myoblasts or exists in other cell types will require future studies. In line with our results, it was reported that in mice lacking p38α in muscle, p38γ is activated and the muscle develops and functions (28). It seems, therefore, that the inhibition of p38γ (and maybe also of p38β) by p38α occurs *in vivo* too.

Notably, p38β, in spite of being so similar to p38α, possesses unique biochemical properties, primarily spontaneous autoactivation generated by a short region composed of the α-G helix and MAPK insert (56). One could speculate that these characteristics may be related to the role of p38β as a backup for p38α. It could be, for example, that under conditions that compromise p38 activation by upstream components, the ability of p38β to autoactivate allows it to take over the roles of other isoforms, mainly of p38α.

In other families of enzymes, the rationale behind the development of several isoforms seems quite clear, and in most cases, they do not serve as a backup for each other. In many cases, enzyme isoforms differ in properties such as affinity to substrates (denoted in Km) and catalysis efficiency (57, 58, 59). These properties were selected in evolution to meet specific metabolic requirements of the tissue in which a given isoform is expressed, or for improved adaptation to changing conditions.

Although both p38β and p38γ can each compensate for the absence of p38α in C2C12 cells, the compensation of p38γ is not comprehensive, reflected in the fact that C2p38α/β^−/−^ cells do not fuse. The p38α + p38β subgroup of p38 molecules is thus critical for full-scale myoblast to myotube differentiation and each kinase could fully compensate for the absence of the other. Expression of myomaker and myomerger requires activity of either p38α or p38β, which cannot be replaced by p38γ. p38γ differs in structural and pharmacological properties from p38α and p38β, and probably does not recognize some of their substrates (20). Yet, as p38γ does activate the myogenic program, the overall conclusion is that p38 activity is essential for C2C12 differentiation and that each of the three isoforms can support it.

The observation that p38γ and p38β are activated and MKK6 is upregulated and phosphorylated in C2p38α^−/−^ suggests that p38α is an inhibitor of p38γ and p38β as well as of its own phosphorylation. A possible simple mechanism for this inhibition may be that p38 isoforms compete for the lower levels of the upstream activators. MKK6 was indeed reported to be a more efficient kinase for p38α than to p38β and p38γ (60). p38α-dependent inhibition of p38γ or p38β may also be achieved through a direct phosphorylation, as p38α is capable of phosphorylating p38β on Ser143, Thr241, and Ser261, and p38γ on Thr241 (61).

Previous observations, many obtained in C2C12 cells, suggested that p38α is critical for the myoblast to myotube process (11, 62, 63). Some studies suggest an important role for p38β and p38γ as well (11, 16). These previous studies relied on short-term treatment with pharmacological inhibitors and siRNA-based downregulation (17, 34). Most probably, these conditions did not provide sufficient time for rewiring the biochemical networks to elevate level and activity of MKK6 and in turn activation of p38β and p38γ. Our experimental model involves stable myogenic cell lines in which p38α was permanently knocked out. These lines proliferate for generations, providing sufficient time for altering their biochemical web, adjusting it to the absence of p38α. Indeed, when the p38 inhibitors are provided to C2C12 cells for a longer period, the response is similar to that observed in C2p38α−/− cells and some degree of differentiation does occur (Fig. 9).

MyoD expression is high and constant in C2p38α/γ/β^−/−^ cells, as opposed to its kinetics in parental C2C12 cells, where its level increases in response to a differentiation signal and is then decreased. Although p38α/β have been shown to positively regulate MyoD through MK2 activity (64), there have been observations of MyoD overexpression in myogenic cells in which p38α or myogenin were knocked out (11, 65). Thus, it is possible that in normal differentiation, some kind of p38-dependent negative feedback is activated and downregulates MyoD expression in late stages of differentiation. This putative mechanism would not be active when p38α, p38β, and p38γ are all missing, explaining the constant MyoD expression.

To promote myogenic differentiation, cells are required to withdraw from the cell cycle, *via* downregulation of components such as cyclin D1 (10). Previous studies showed that ectopic expression of cyclin D1 prevents cell cycle arrest and thus differentiation (10). In addition, a primary myoblast culture from mice-deficient p38α shows an increase in cyclin D1 (39). We found high expression levels in C2p38α/β^−/−^ cells and, more significantly, in C2p38α/γ/β^−/−^ cells, which can explain the high percentage of cells in S phase, as well as foci formation in C2p38α/γ/β^−/−^ cells. Our results indicate that the activity of p38α/β is essential for cyclin D1 downregulation in myoblast to myotubes differentiation. p38γ does not perform this function.

In summary, this study demonstrated that p38 activity is necessary for the myoblast to myotube differentiation, but no single isoform is truly indispensable. We propose that the relationships between the isoforms were developed during evolution in a way that enables compensatory activity of other isoforms in the absence of one of them. This mechanism, which secures execution of the physiological function of the p38 family, may not be specific to the myogenic system, but could occur in the many other cell lineages.

## Experimental procedures

### Growth of C2C12 and derivatives

C2C12 cells (13) were grown in growth medium (GM), composed of Dulbecco's modified Eagle's medium (DMEM) supplemented with 10% fetal bovine serum, Na-pyruvate, and antibiotics. Cells were grown at 37 °C under 5% CO_2_. To induce differentiation, C2C12 and its derivatives, which were constructed in the course of this study, C2p38α^−/−^, C2p38β^−/−^, C2p38γ^−/−^, C2p38α/γ^−/−^, C2p38β/γ^−/−^, and C2p38α/β/γ ^−/−^ were grown to a subconfluence concentration and then GM was replaced with DM, composed of DMEM supplemented with 2% horse serum, Na-pyruvate, 0.1 μg/ml insulin (Sigma-Aldrich, I1882), and antibiotics.

RD cells were grown in GM, composed of DMEM supplemented with 10% fetal bovine serum, Na-pyruvate, and antibiotics. Cells were grown at 37 °C under 5% CO_2_.

### Generating various C2C12 KO cells

Cells were seeded at 1 × 10^6^/10 cm plate for 24 h and were then co-transfected with CRISPR/Cas9 KO plasmids (include three different single guide RNAs [sgRNAs]) using Mirus TransIT-X2 (MIR6004) according to the manufacturer’s instructions. Cells were sorted for GFP-positive cells 48 h after transfection using Beckman Coulter *Moflo Astrios* flow cytometer-sorter equipped with four lasers (405, 488, 561, and 640 nm). All flow cytometry data were analyzed with FACSDiva software (BD Biosciences; https://www.bdbiosciences.com/content/dam/bdb/marketing-documents/eu/23-9549-01_FACSDivas8-Qrg-FACSCanto-HTS.pdf). Fluorophores were detected using the default detector arrays supplied with the instrument. Voltage settings for side scatter, forward scatter, and fluorescence channels were kept constant for all experiments described. GFP-positive cells were sorted using a 100 μm nozzle and minimal sorting efficiency of 90%.

Positive cells were seeded as single cells in 96-well plate supplemented with GM. Finally, clones were harvested, and lysates tested to identify KO clones using the appropriate antibodies. C2p38α^−/−^ cells were produced using a p38α CRISPR/Cas9 KO plasmid (Santa Cruz, sc-424051). C2p38γ^−/−^ and C2p38α/γ^−/−^ cells were produced using three different p38γ CRISPR/Cas9 KO plasmids (GeneCopoeia, MCP229967-CG04-3). C2p38β^−/−^ and C2p38α/γ/β^−/−^ cells were produced using three different p38β CRISPR/Cas9 KO plasmids (GeneCopoeia, GC-MCP229967-CG04-3-B). C2p38β/γ^−/−^ cells were produced using the same p38β and p38γ CRISPR/Cas9 KO plasmids as described.

### Verification of p38**β** knockout in C2C12 and in C2p38**α/γ**^−/−^ cells

Genomic DNA of single cell–based clones which were positive for transfection of CRISPR/Cas9 KO plasmids was extracted using PureLink Genomic DNA Mini Kit (Invitrogen, K182001) according to the manufacturer’s protocol. For PCR amplification of sgRNA-targeting genome regions with the corresponding primers (listed below), Q5 Hot Start High-Fidelity 2X Master Mix (NEB, M0494S) was used, according to the manufacturer's instructions. Then, PCR products were subjected to the following procedures: i) sequencing using the same primers as for amplification and ii) digestion by 1 μl of T7 endonuclease I (NEB, M0302S), according to the manufacturer's instructions, followed by loading of the digestion products on a 2% agarose gel.

Clones which had their amplified sgRNA-targeting DNA region cleaved by T7 endonuclease were selected, as the sequence was disrupted and not aligned with intact C2C12 cells DNA.

Primers for sgRNA-targeting genome regions in p38β gene, used for amplification and sequencing: 5′-CAAGAGAGTAGGTGTCCGC-3′ and 5′-CTTTGCTCAGCGGTGCTG-3′.

### Western blot analysis

Protein lysates of C2C12 and C2C12-derived cells were prepared by decanting the medium, washing the cells with PBS and adding Laemmli buffer directly to the plate. Lysates were then collected and boiled for 10 min. Twenty micrograms of protein lysates were separated *via* a 10% SDS-PAGE and transferred to a nitrocellulose paper. After incubation of the membrane with the appropriate antibodies (see below), specific proteins were visualized using an enhanced chemiluminescence detection reagent.

Antibodies used in this study were: anti-phospho-p38 (Cell Signaling, 9211), anti p38α (Santa Cruz, sc535 and Cell Signaling, 9228S), anti-p38γ (R&D systems, MAB1347), anti-phospho MAPKAPK2 (Cell Signaling, 3007), anti-MAPKAPK2 (Cell Signaling, 3042), anti myogenin (Santa Cruz, sc12732), anti-MyoD (Santa Cruz, sc304), anti-MyHC (obtained from the MF20 hybridoma cell line), anti-Creatine Kinase (Santa Cruz, sc15161), anti-Myomaker (TMEM8C) (Novus, NBP2-94514), anti-Myomerger (ESGP) (Invitrogen,#PA5-47639), and anti-GAPDH (Invitrogen, AM4300).

### p38 inhibitors

BIRB796 was purchased from Calbiochem (506172) and SB203580 from Sigma-Aldrich (S8307).

### Immunofluorescence staining

Cells were fixed with 4% paraformaldehyde for 10 min and permeabilized with 1% Triton X-100 for 4 min prior to labeling. Cells were stained with total MyHC primary antibody (obtained from the MF20 hybridoma cell line) then with Alexa fluor 568 secondary antibody (Abcam AB175701) diluted 1∶300 and nuclei with 1 μg/ml DAPI (BioLegend, 422801) in PBS for 10 min.

### Microscopy

Light microscope images were attained using an Olympus IX70 microscope with a 10X/0.3 objective and Sony 2.3 MP, mono IMX174, IDS camera. Confocal laser scanning microscopy was performed using an FV-1000 confocal workstation (Olympus), with 20X/0.75 objective.

### Observation of foci

1 × 10^6^ C2C12, C2p38α/β^−/−^, C2p38α/β/γ^−/−^, and RD cells were seeded and left to grow for 18 days in GM. Images of foci were taken at day 18.

### Cell cycle analysis

1 × 10^6^ C2C12 and C2p38α/β^−/−^, C2 p38α/β/γ^−/−^, and RD cells were washed twice with PBS then incubated at 4 °C for 30 min in 2 ml of 70% ethanol for fixation purposes and to minimize clumping. Next, cells were washed with 2 ml PBS and treated with 50 μl of 100 μg/ml RNase A for 30 min at 37 °C to remove RNA traces from the samples. Two hundred microliters of 50 μg/ml propidium iodide (Sigma-Aldrich, P4864) was added. Flow cytometry analysis was performed using a CellStream analyser flow cytometer sorter (BD Biosciences; https://www.bdbiosciences.com/en-eu/products/instruments/flow-cytometers) equipped with 4 lasers (405, 488, 561, 633 nm). Fluorophores were detected using the default detector arrays supplied with the instrument. Voltage settings for side scatter, forward scatter, and fluorescence channels were kept constant for all experiments described. Data were recorded for ≥10,000 cells from each sample and data were analyzed with FACSDiva software (BD Biosciences).

## Data availability

All data are contained within the article.

## Supporting information

This article contains supporting information.

## Conflict of interest

The authors declare that they have no conflicts of interest with the contents of this article.
