# Supplementary material for: Knocking out p38α+p38β+p38γ is required to abort the myogenic program in C2C12 myoblasts and to impose uncontrolled proliferation
Source: J Biol Chem. 2025 Feb 6;301(3):108281. doi: 10.1016/j.jbc.2025.108281 (PMC11925101; doi:10.1016/j.jbc.2025.108281)
Supplement: Supplementary Figures [file mmc1.pptx]

## Slide 1
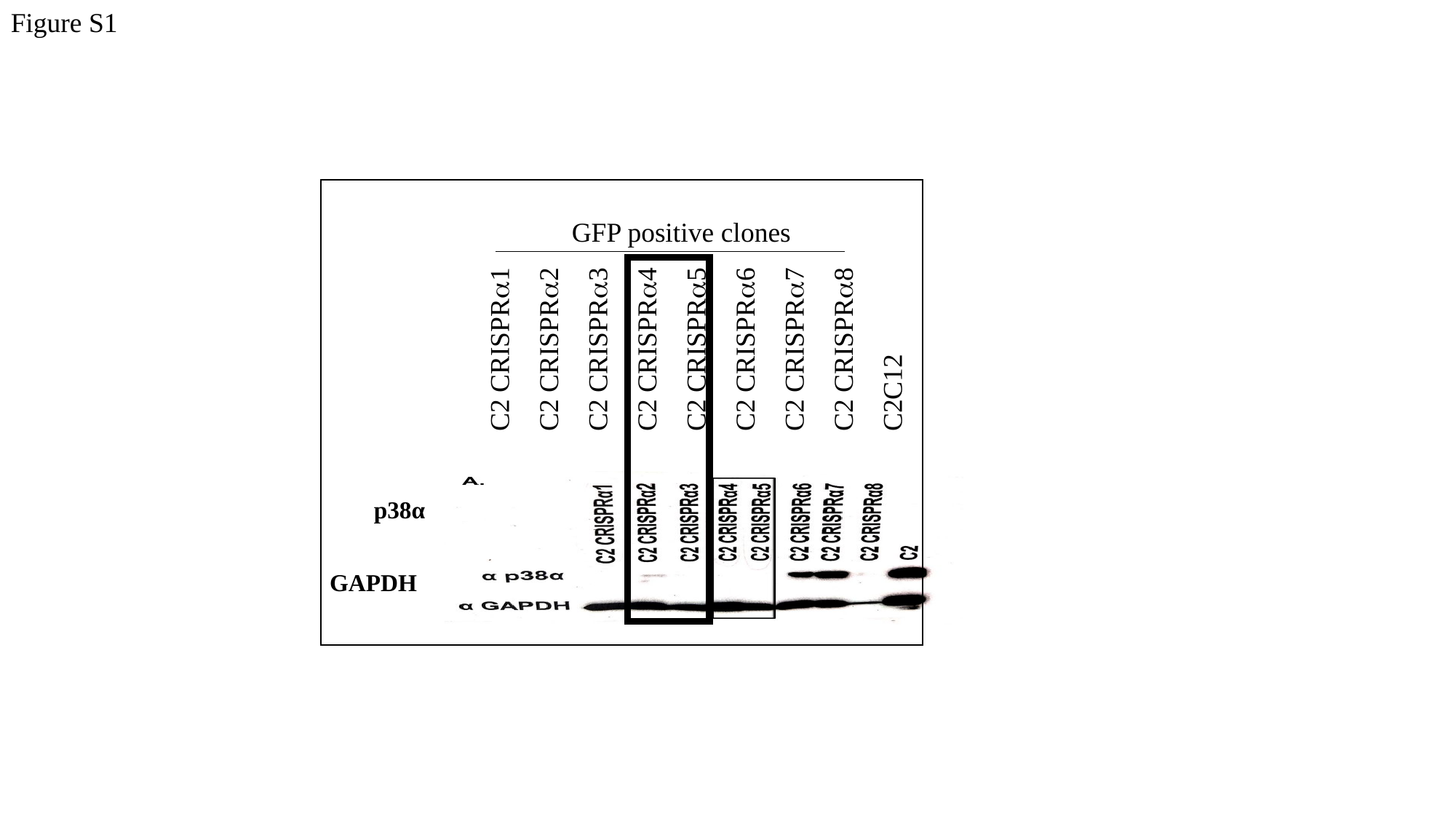

Figure S1
GFP positive clones
C2 CRISPRa1
C2 CRISPRa2
C2 CRISPRa3
C2 CRISPRa4
C2 CRISPRa5
C2 CRISPRa6
C2 CRISPRa7
C2 CRISPRa8
C2C12
p38α
GAPDH

## Slide 2
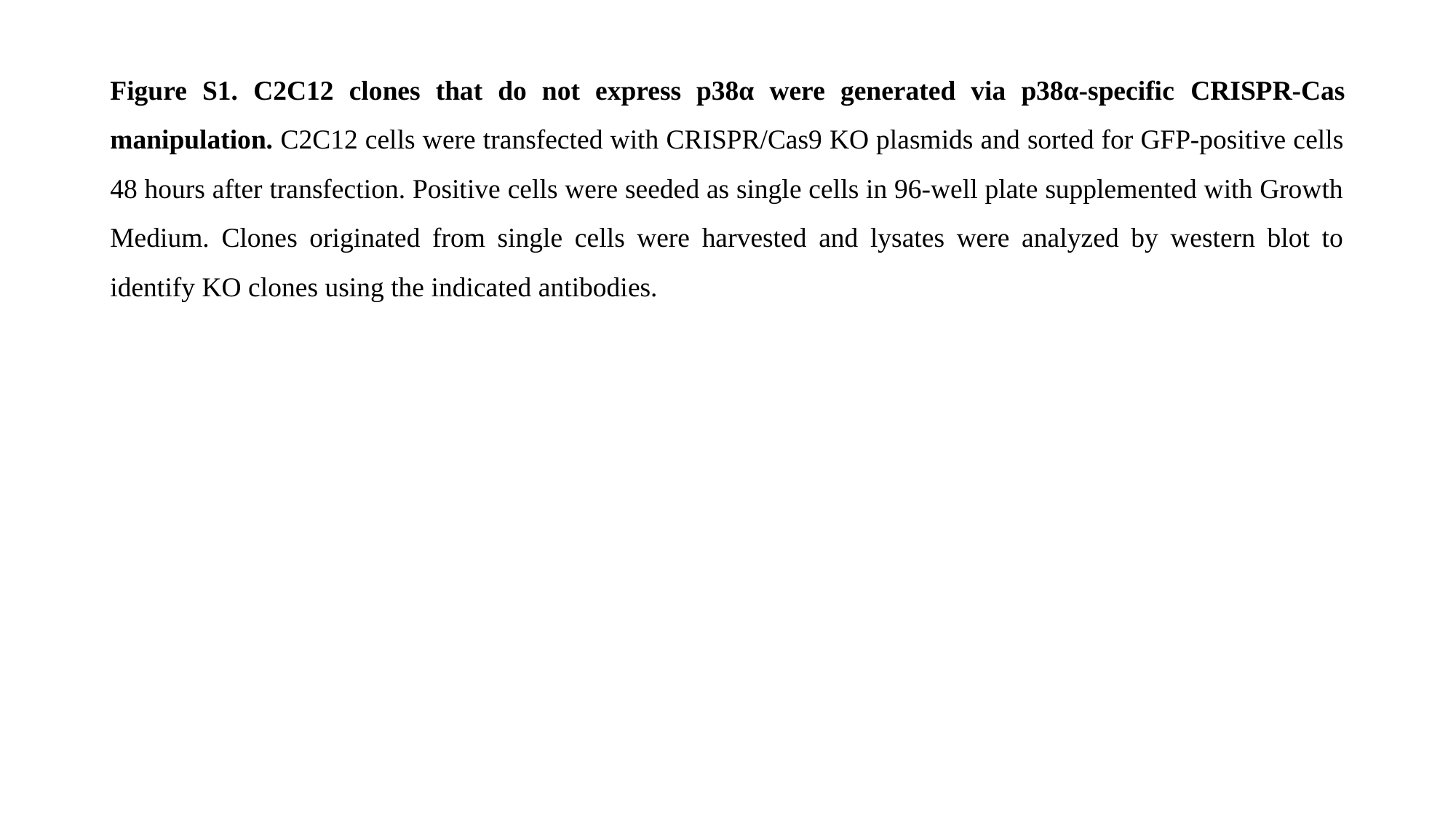

Figure S1. C2C12 clones that do not express p38α were generated via p38α-specific CRISPR-Cas manipulation. C2C12 cells were transfected with CRISPR/Cas9 KO plasmids and sorted for GFP-positive cells 48 hours after transfection. Positive cells were seeded as single cells in 96-well plate supplemented with Growth Medium. Clones originated from single cells were harvested and lysates were analyzed by western blot to identify KO clones using the indicated antibodies.

## Slide 3
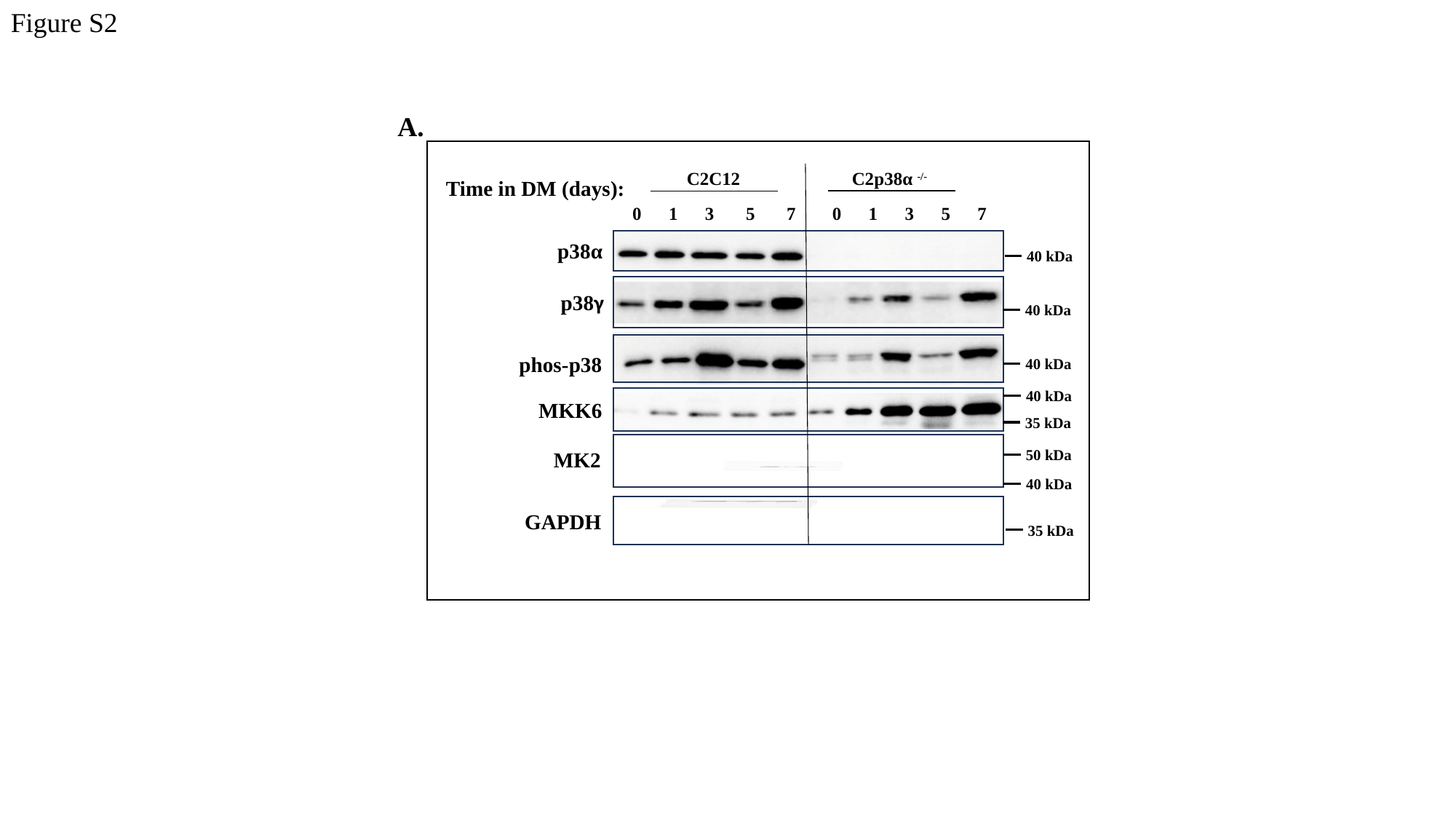

Figure S2
A.
 C2C12
C2p38α -/-
Time in DM (days):
0 1 3 5 7 0 1 3 5 7
p38α
40 kDa
p38γ
40 kDa
 phos-p38
40 kDa
40 kDa
MKK6
35 kDa
50 kDa
MK2
40 kDa
GAPDH
35 kDa

## Slide 4
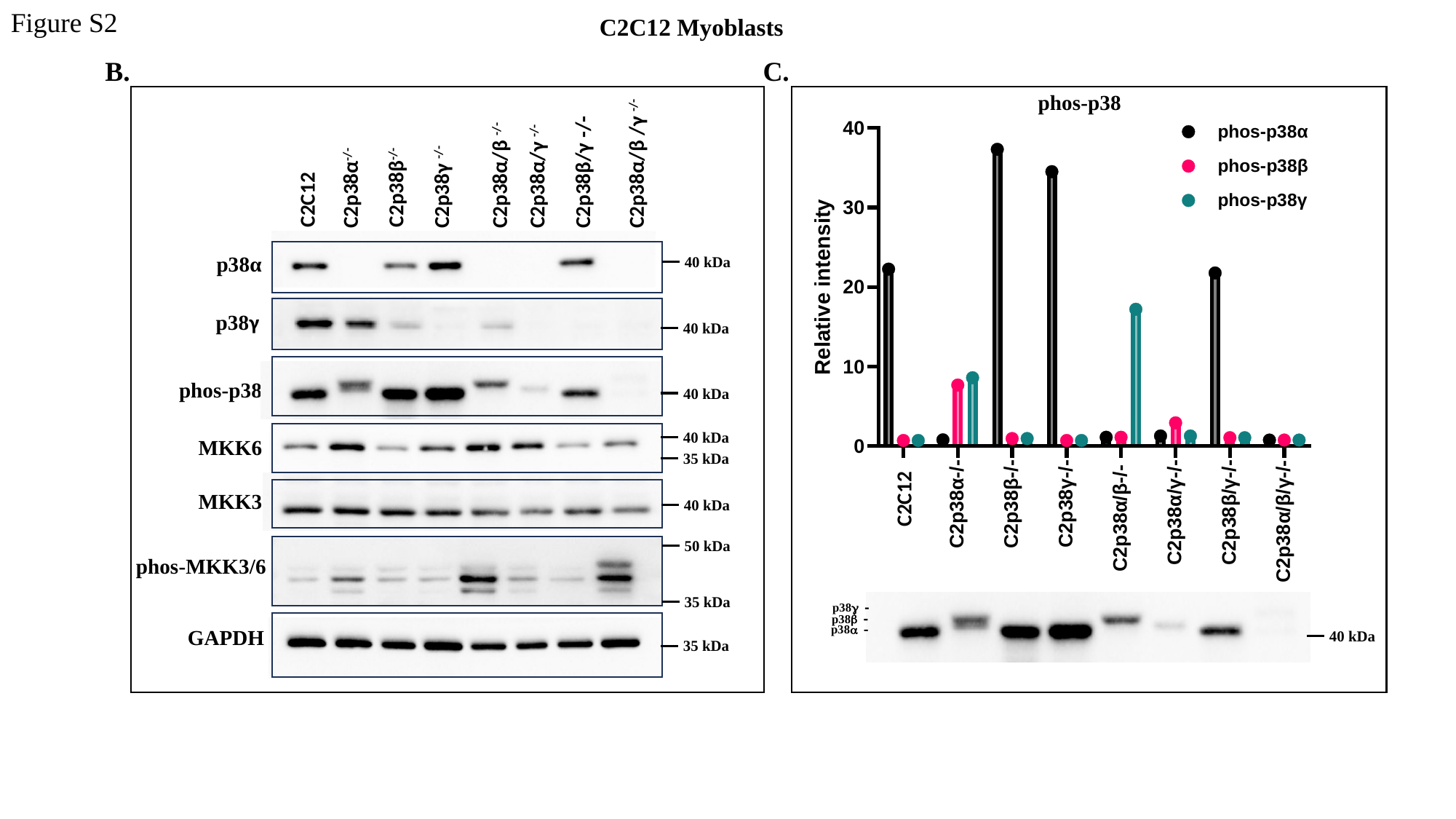

Figure S2
C2C12 Myoblasts
B.
C.
phos-p38
C2p38α/β /γ -/-
C2p38α/γ -/-
C2p38β/γ -/-
C2p38γ -/-
C2p38α/β -/-
C2p38β-/-
C2p38α-/-
C2C12
p38α
40 kDa
p38γ
40 kDa
 phos-p38
40 kDa
40 kDa
MKK6
35 kDa
C2C12
MKK3
40 kDa
50 kDa
phos-MKK3/6
35 kDa
p38g -
p38b -
p38a -
GAPDH
40 kDa
35 kDa

## Slide 5
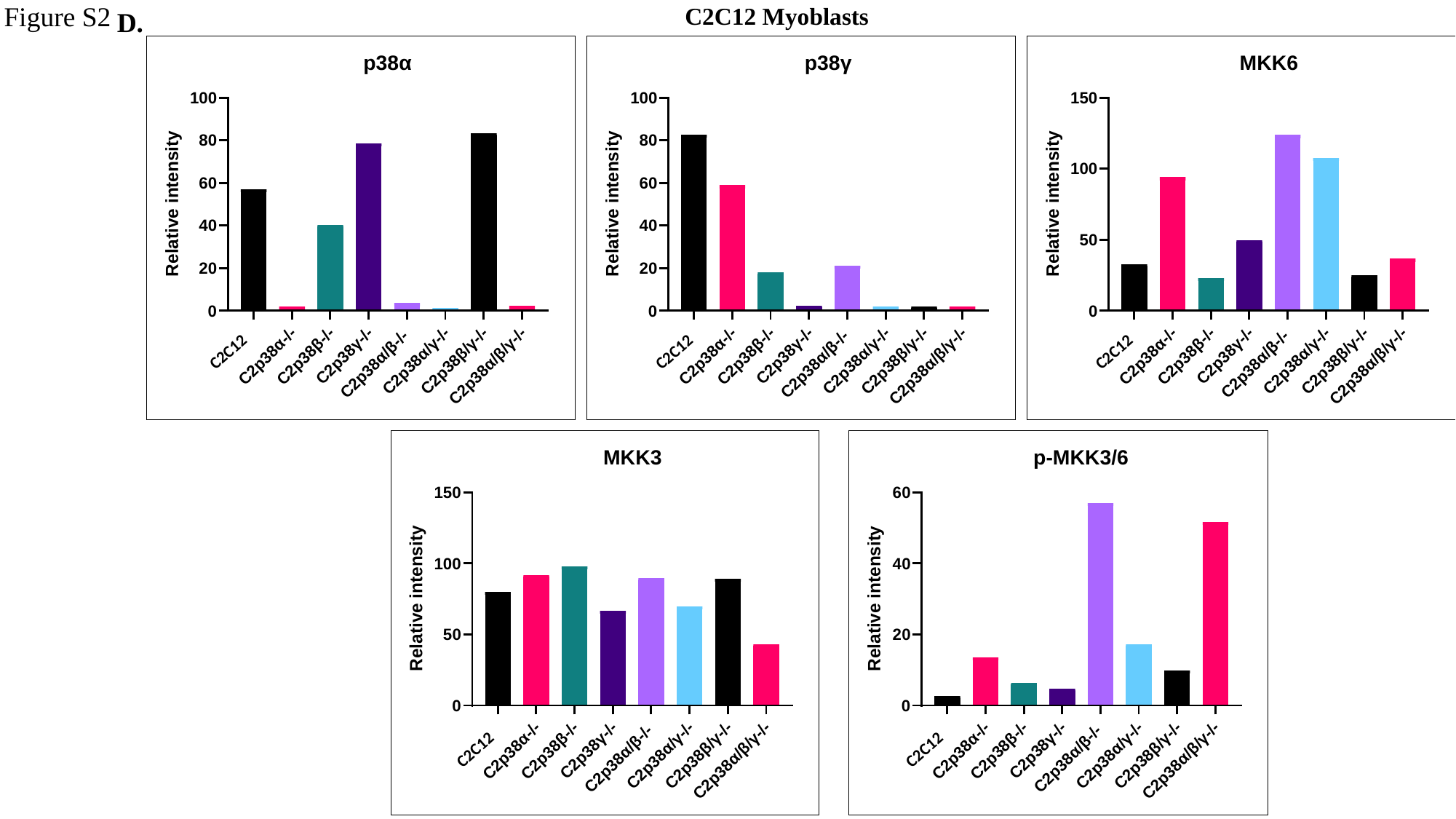

D.
Figure S2
C2C12 Myoblasts
C2C12
C2C12
C2C12
C2C12
C2C12

## Slide 6
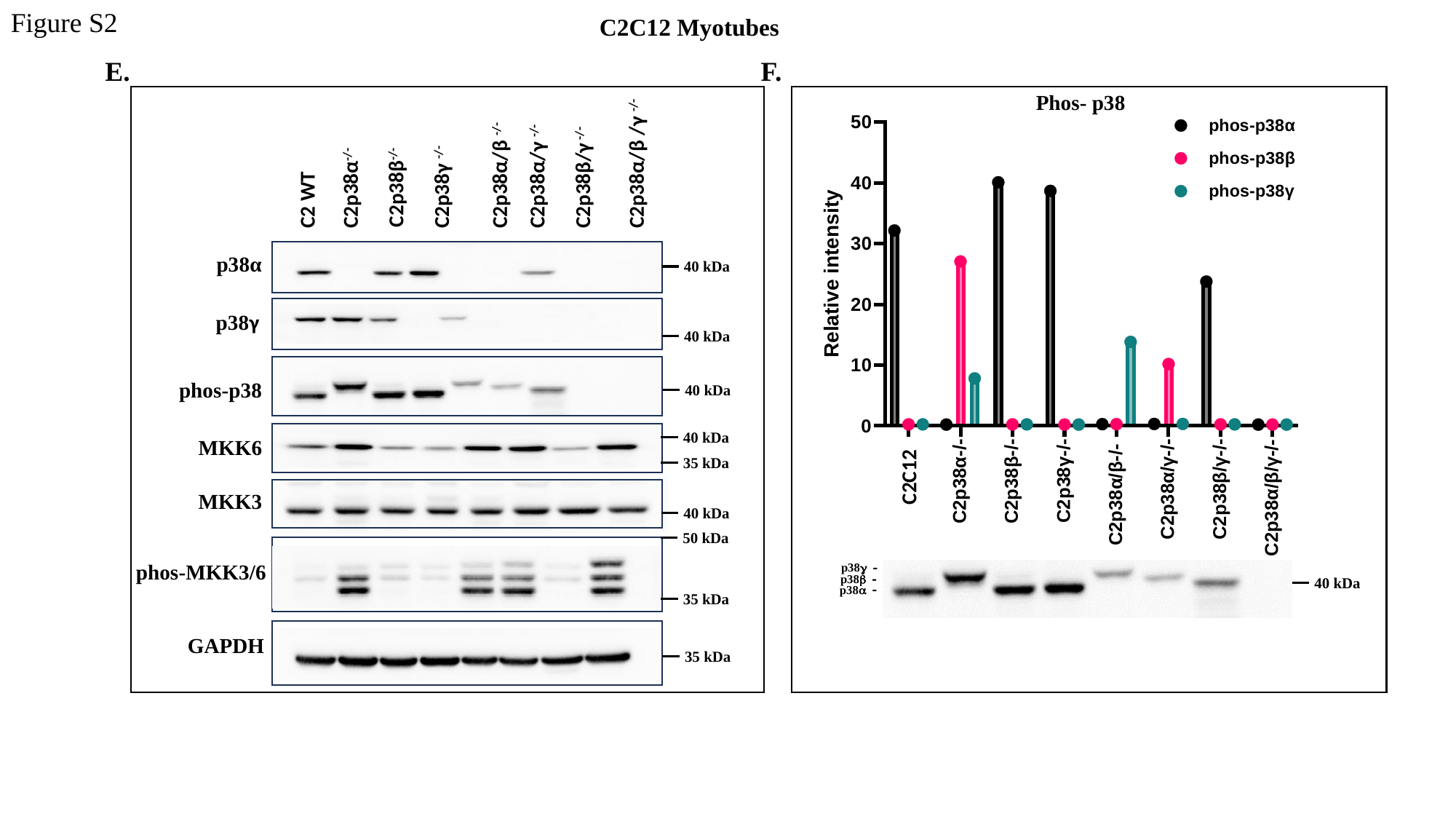

Figure S2
C2C12 Myotubes
E.
F.
Phos- p38
C2p38α/β /γ -/-
C2p38α/γ -/-
C2p38β/γ -/-
C2p38γ -/-
C2p38α/β -/-
C2p38β-/-
C2p38α-/-
C2 WT
p38α
40 kDa
p38γ
40 kDa
 phos-p38
40 kDa
40 kDa
MKK6
35 kDa
C2C12
MKK3
40 kDa
50 kDa
phos-MKK3/6
p38g -
p38b -
40 kDa
p38a -
35 kDa
GAPDH
35 kDa

## Slide 7
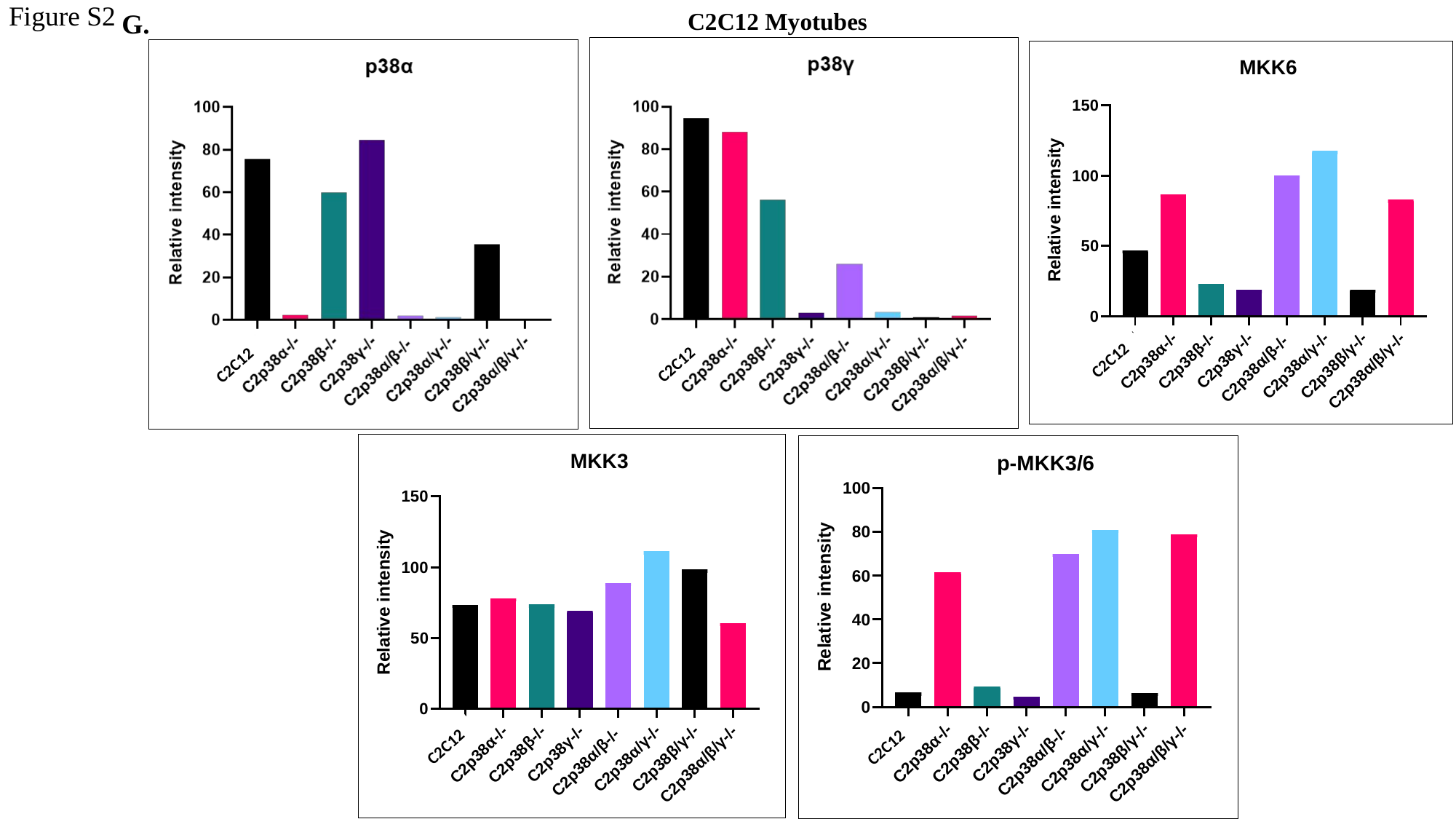

Figure S2
C2C12 Myotubes
G.
C2C12
C2C12
C2C12
C2C12
C2C12

## Slide 8
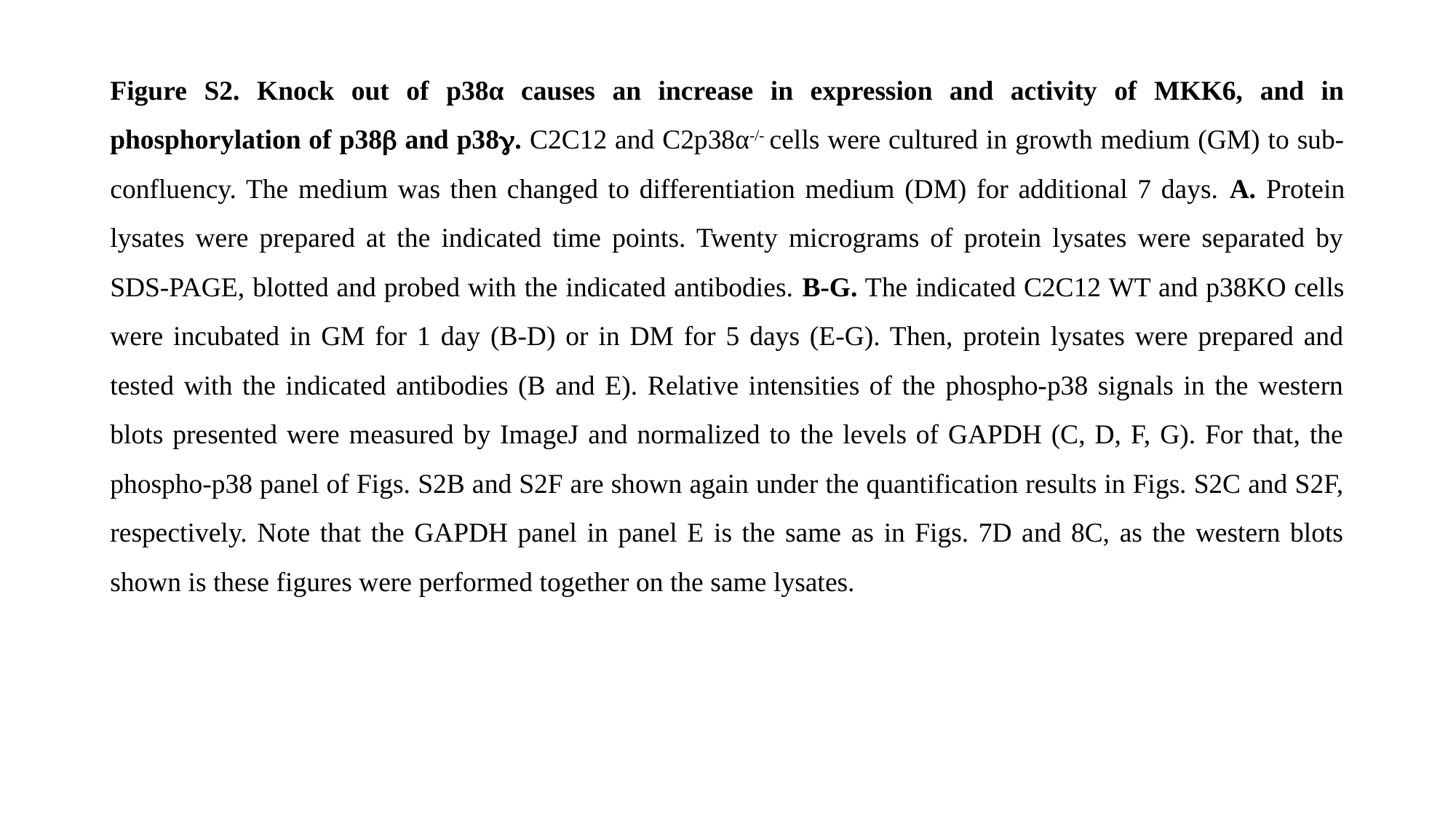

Figure S2. Knock out of p38α causes an increase in expression and activity of MKK6, and in phosphorylation of p38b and p38g. C2C12 and C2p38α-/- cells were cultured in growth medium (GM) to sub-confluency. The medium was then changed to differentiation medium (DM) for additional 7 days. A. Protein lysates were prepared at the indicated time points. Twenty micrograms of protein lysates were separated by SDS-PAGE, blotted and probed with the indicated antibodies. B-G. The indicated C2C12 WT and p38KO cells were incubated in GM for 1 day (B-D) or in DM for 5 days (E-G). Then, protein lysates were prepared and tested with the indicated antibodies (B and E). Relative intensities of the phospho-p38 signals in the western blots presented were measured by ImageJ and normalized to the levels of GAPDH (C, D, F, G). For that, the phospho-p38 panel of Figs. S2B and S2F are shown again under the quantification results in Figs. S2C and S2F, respectively. Note that the GAPDH panel in panel E is the same as in Figs. 7D and 8C, as the western blots shown is these figures were performed together on the same lysates.

## Slide 9
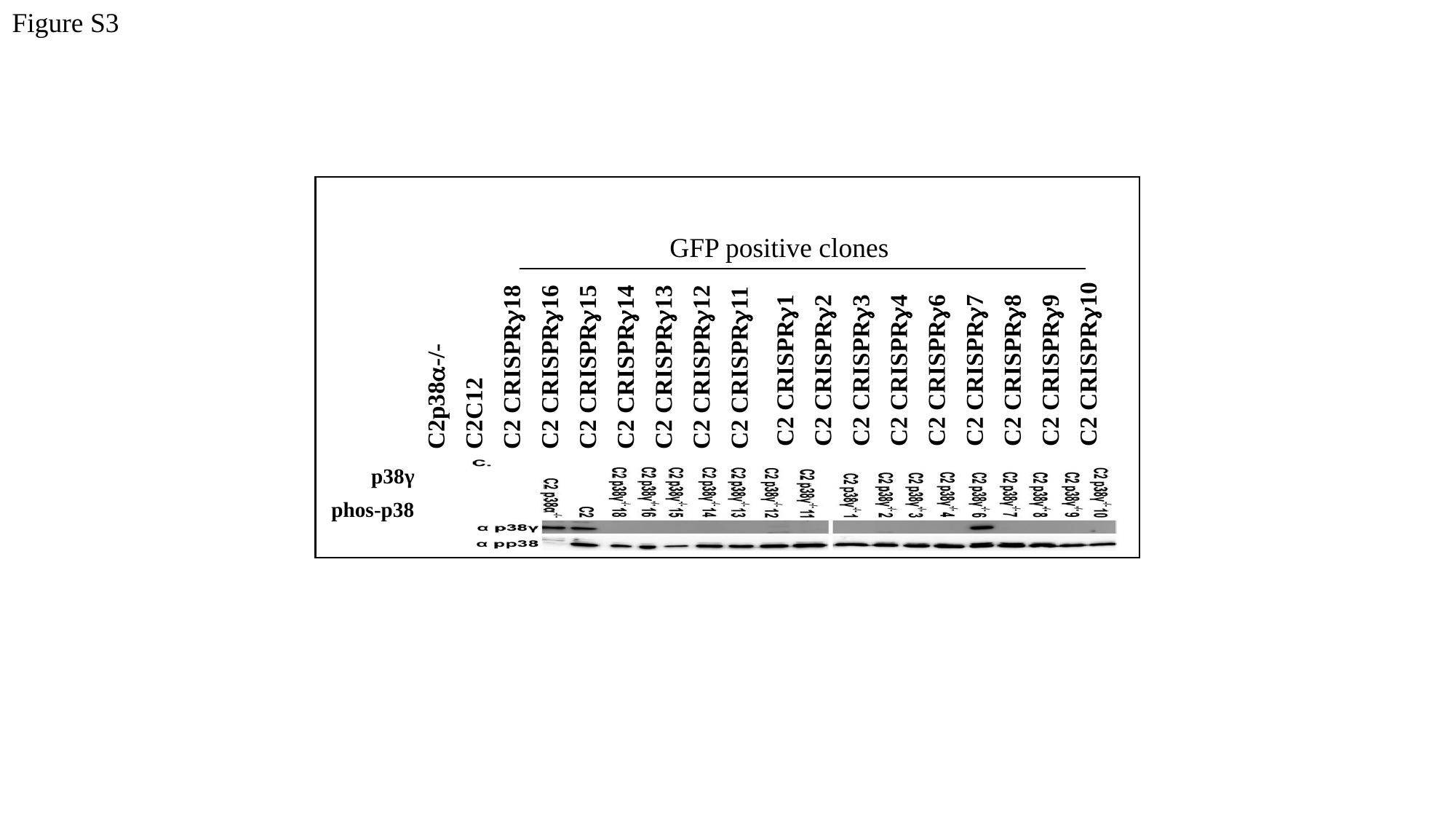

Figure S3
GFP positive clones
C2 CRISPRg1
C2 CRISPRg2
C2 CRISPRg3
C2 CRISPRg4
C2 CRISPRg6
C2 CRISPRg7
C2 CRISPRg8
C2 CRISPRg9
C2 CRISPRg10
C2p38a-/-
C2C12
C2 CRISPRg18
C2 CRISPRg16
C2 CRISPRg15
C2 CRISPRg14
C2 CRISPRg13
C2 CRISPRg12
C2 CRISPRg11
p38γ
 phos-p38

## Slide 10
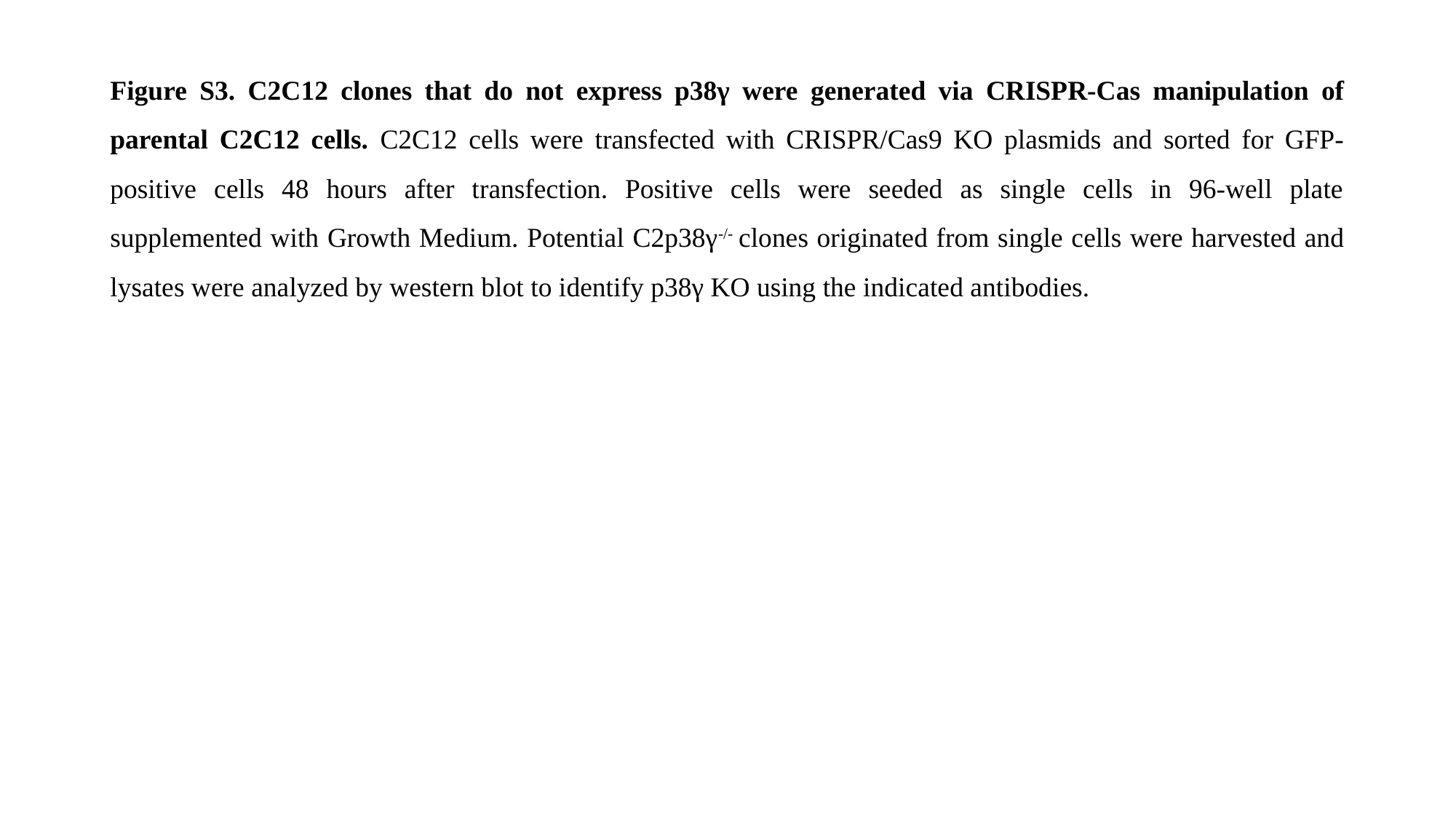

Figure S3. C2C12 clones that do not express p38γ were generated via CRISPR-Cas manipulation of parental C2C12 cells. C2C12 cells were transfected with CRISPR/Cas9 KO plasmids and sorted for GFP-positive cells 48 hours after transfection. Positive cells were seeded as single cells in 96-well plate supplemented with Growth Medium. Potential C2p38γ-/- clones originated from single cells were harvested and lysates were analyzed by western blot to identify p38γ KO using the indicated antibodies.

## Slide 11
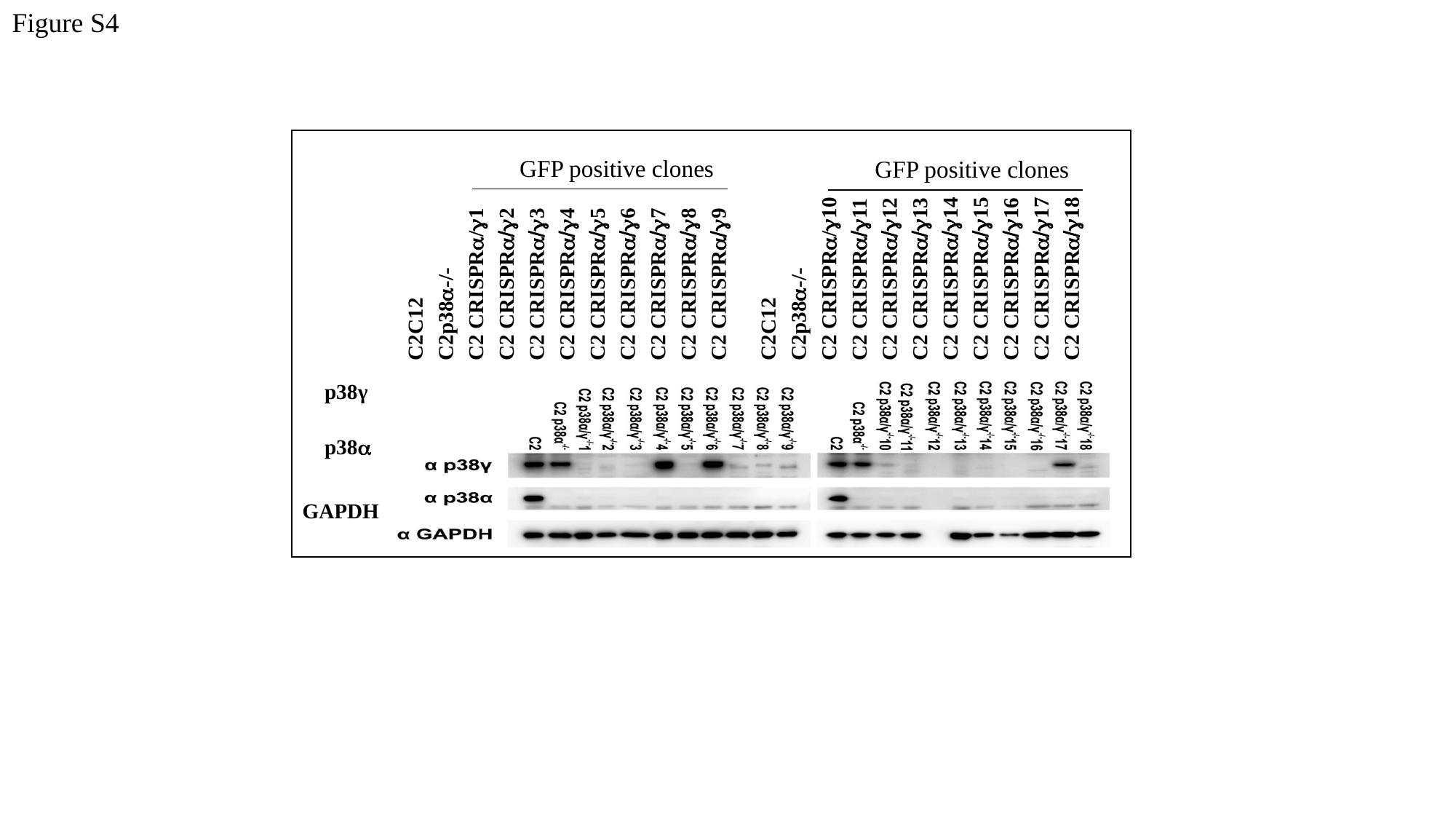

Figure S4
GFP positive clones
GFP positive clones
C2C12
C2p38a-/-
C2 CRISPRa/g10
C2 CRISPRa/g11
C2 CRISPRa/g12
C2 CRISPRa/g13
C2 CRISPRa/g14
C2 CRISPRa/g15
C2 CRISPRa/g16
C2 CRISPRa/g17
C2 CRISPRa/g18
C2C12
C2p38a-/-
C2 CRISPRa/g1
C2 CRISPRa/g2
C2 CRISPRa/g3
C2 CRISPRa/g4
C2 CRISPRa/g5
C2 CRISPRa/g6
C2 CRISPRa/g7
C2 CRISPRa/g8
C2 CRISPRa/g9
p38γ
p38a
GAPDH

## Slide 12
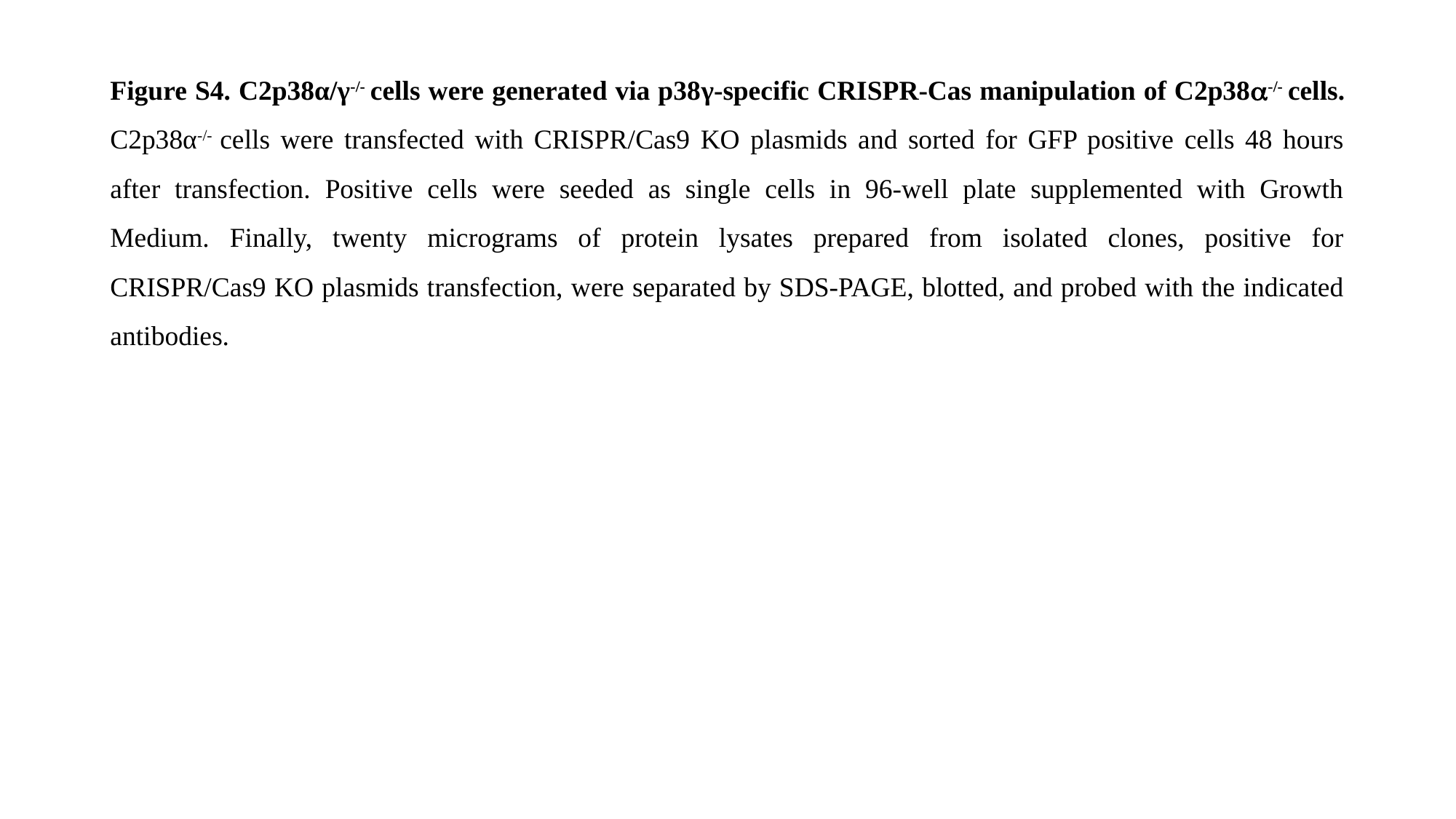

Figure S4. C2p38α/γ-/- cells were generated via p38γ-specific CRISPR-Cas manipulation of C2p38a-/- cells. C2p38α-/- cells were transfected with CRISPR/Cas9 KO plasmids and sorted for GFP positive cells 48 hours after transfection. Positive cells were seeded as single cells in 96-well plate supplemented with Growth Medium. Finally, twenty micrograms of protein lysates prepared from isolated clones, positive for CRISPR/Cas9 KO plasmids transfection, were separated by SDS-PAGE, blotted, and probed with the indicated antibodies.

## Slide 13
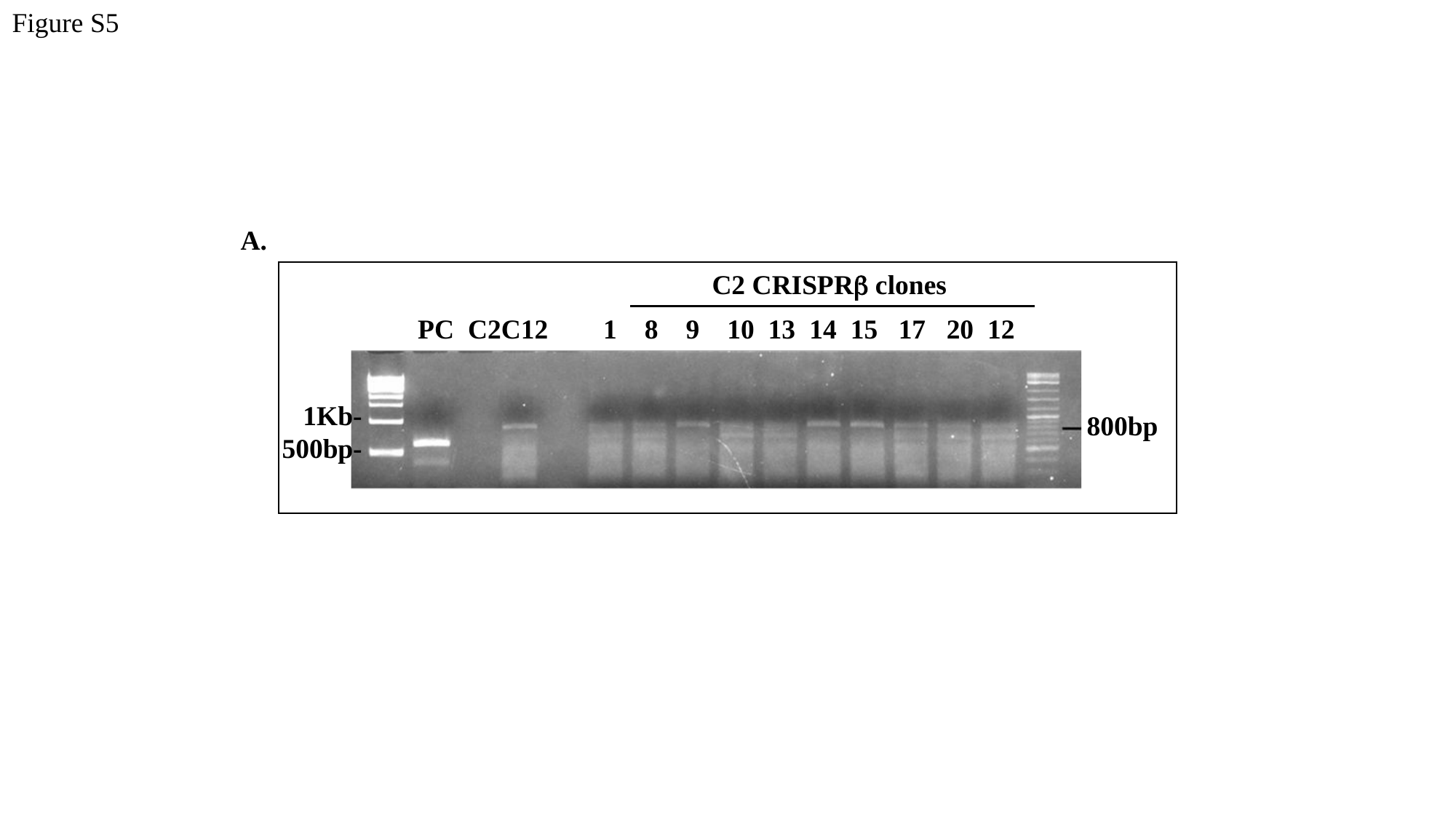

Figure S5
A.
C2 CRISPRb clones
 PC C2C12 1 8 9 10 13 14 15 17 20 12
1Kb-
 500bp-
800bp

## Slide 14
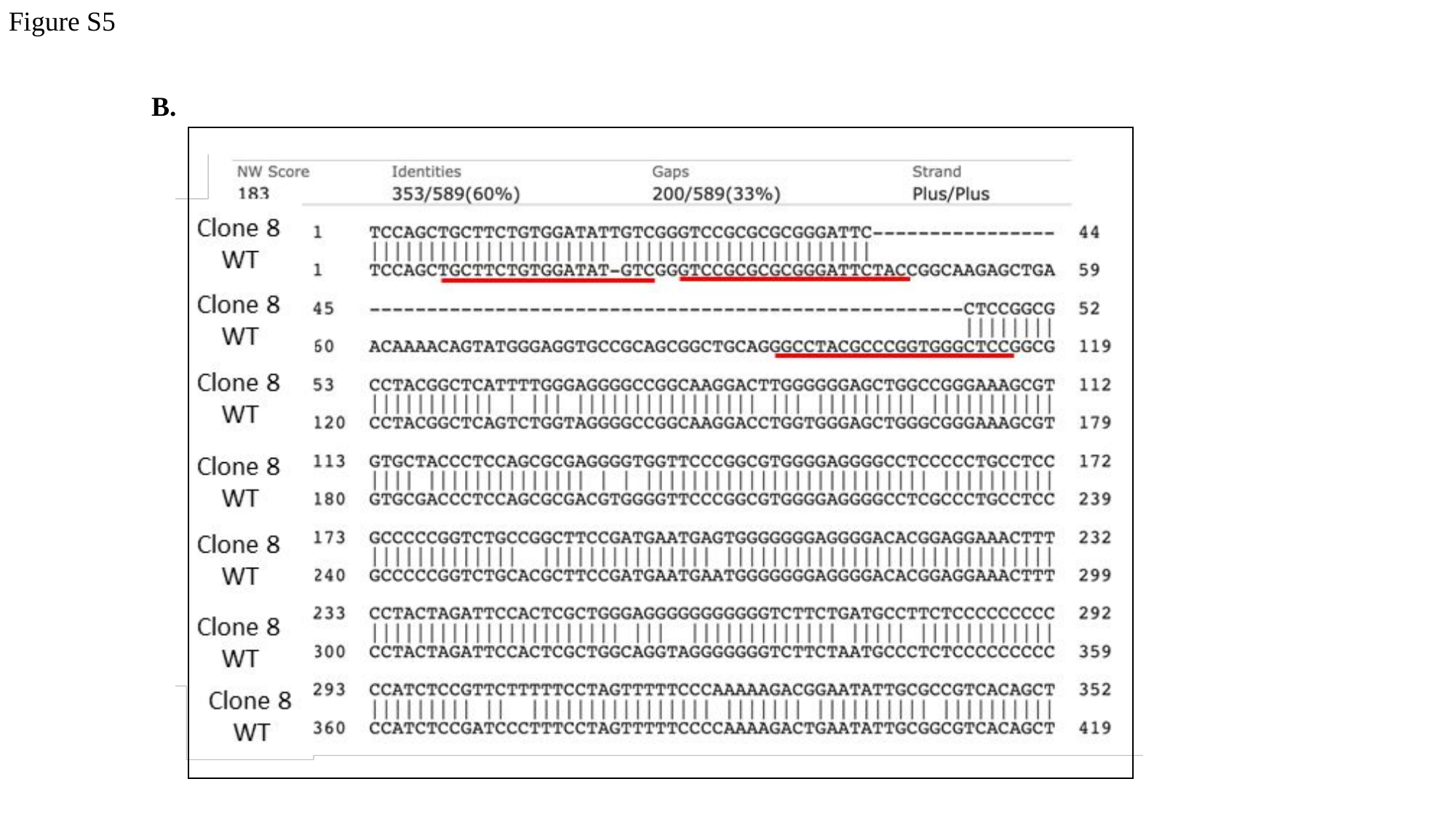

Figure S5
B.

## Slide 15
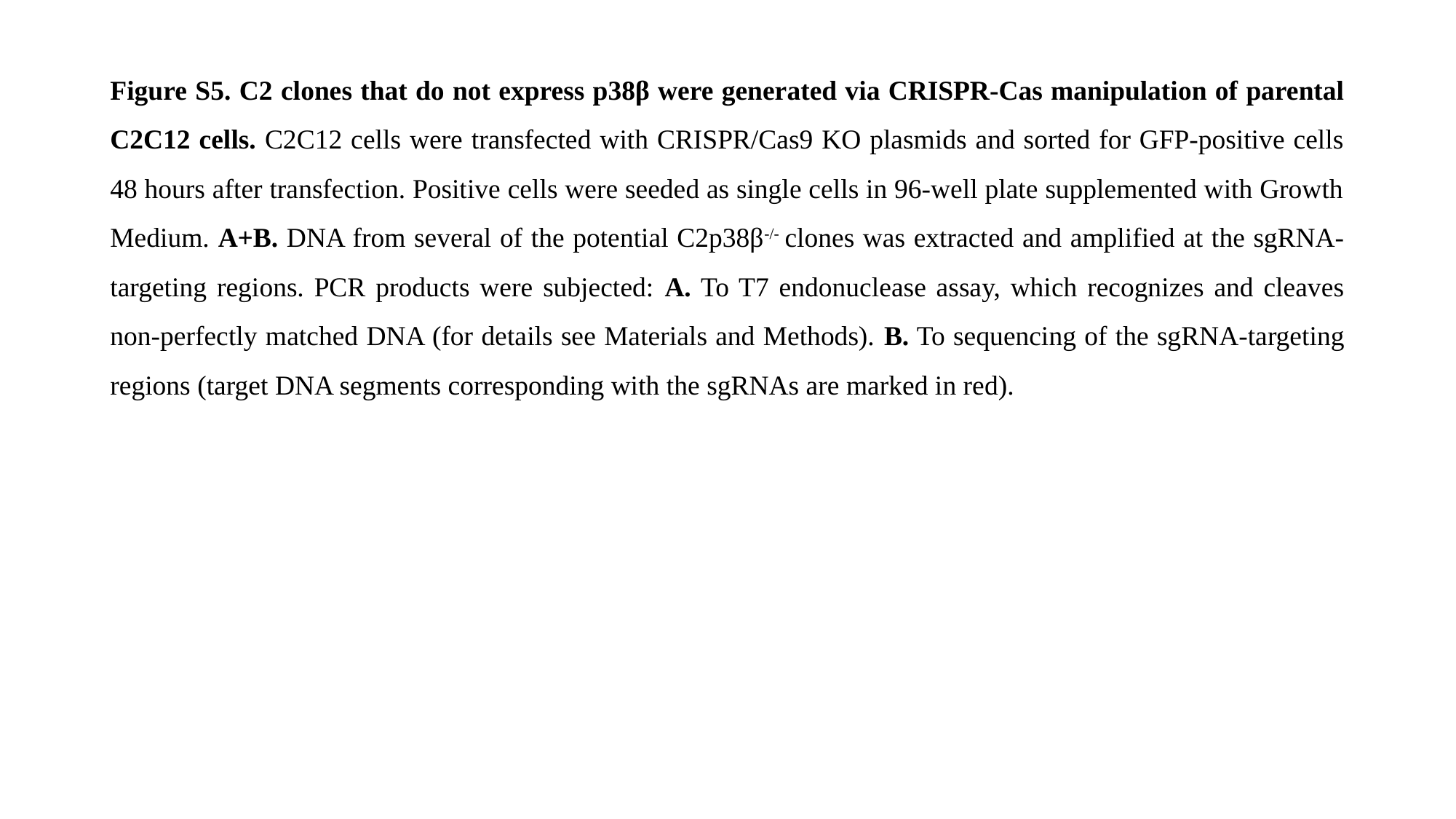

Figure S5. C2 clones that do not express p38β were generated via CRISPR-Cas manipulation of parental C2C12 cells. C2C12 cells were transfected with CRISPR/Cas9 KO plasmids and sorted for GFP-positive cells 48 hours after transfection. Positive cells were seeded as single cells in 96-well plate supplemented with Growth Medium. A+B. DNA from several of the potential C2p38β-/- clones was extracted and amplified at the sgRNA-targeting regions. PCR products were subjected: A. To T7 endonuclease assay, which recognizes and cleaves non-perfectly matched DNA (for details see Materials and Methods). B. To sequencing of the sgRNA-targeting regions (target DNA segments corresponding with the sgRNAs are marked in red).

## Slide 16
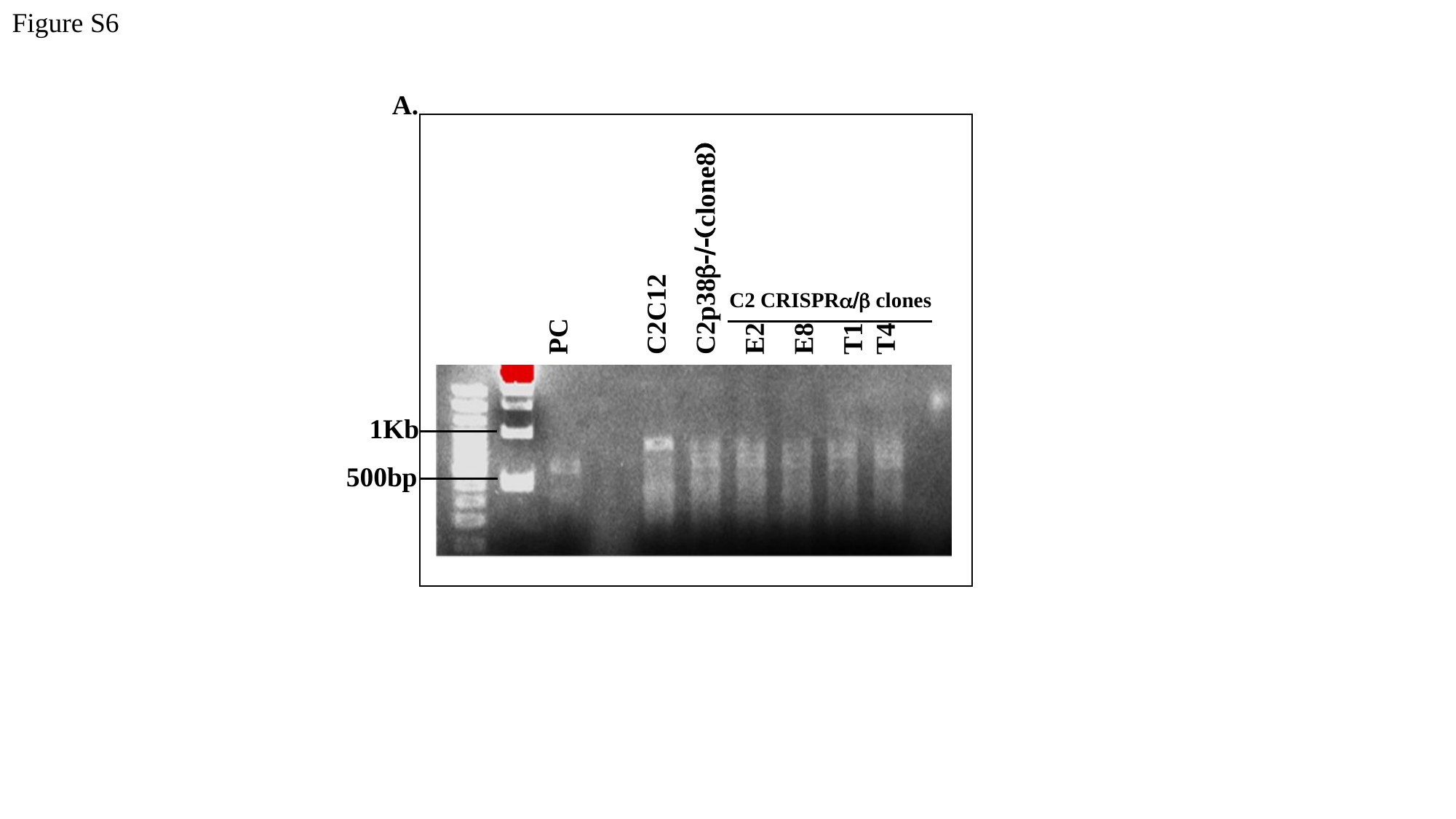

Figure S6
A.
PC
C2C12
C2p38b-/-(clone8)
E2
E8
T1
T4
C2 CRISPRa/b clones
1Kb
500bp

## Slide 17
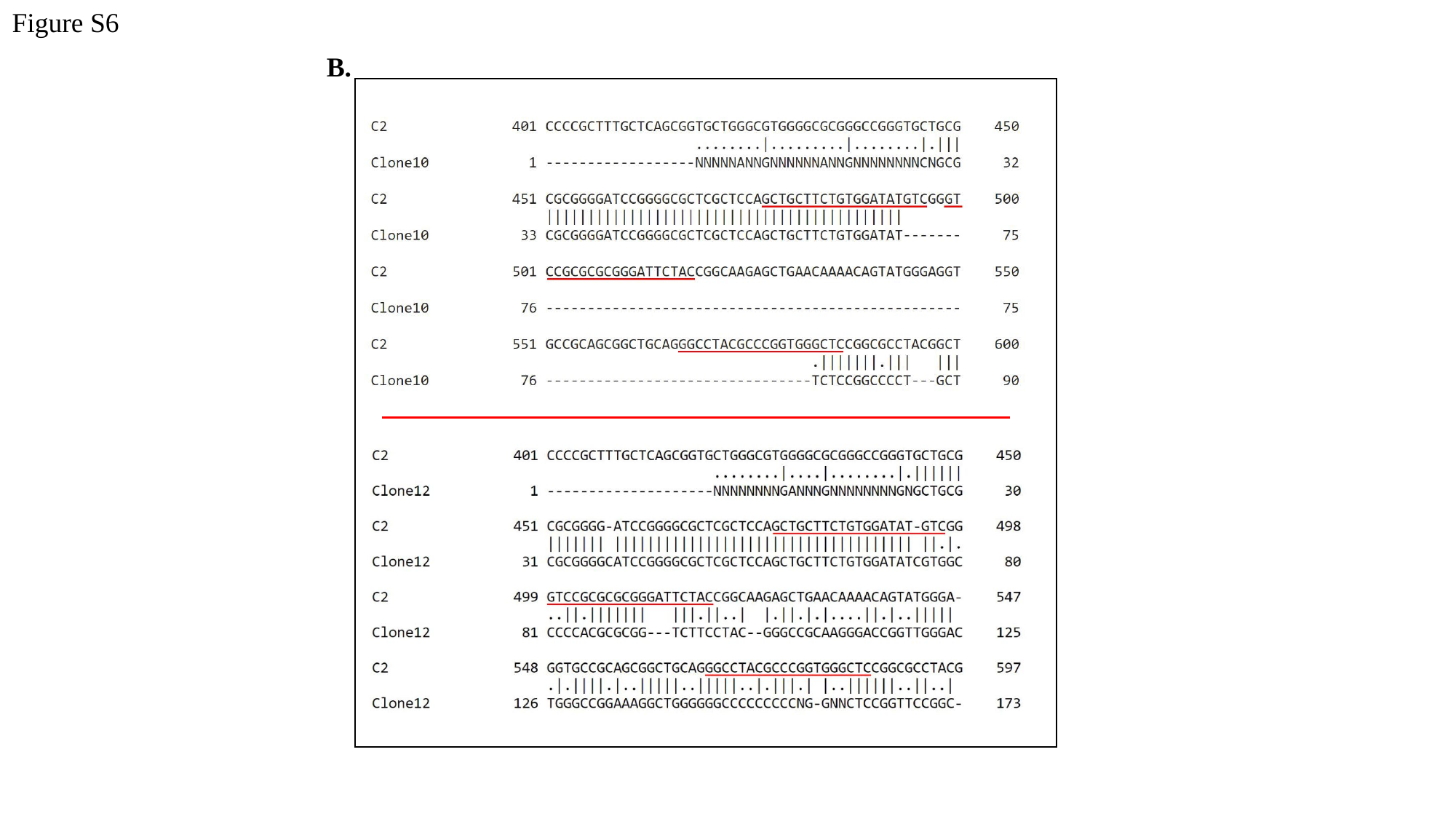

Figure S6
B.

## Slide 18
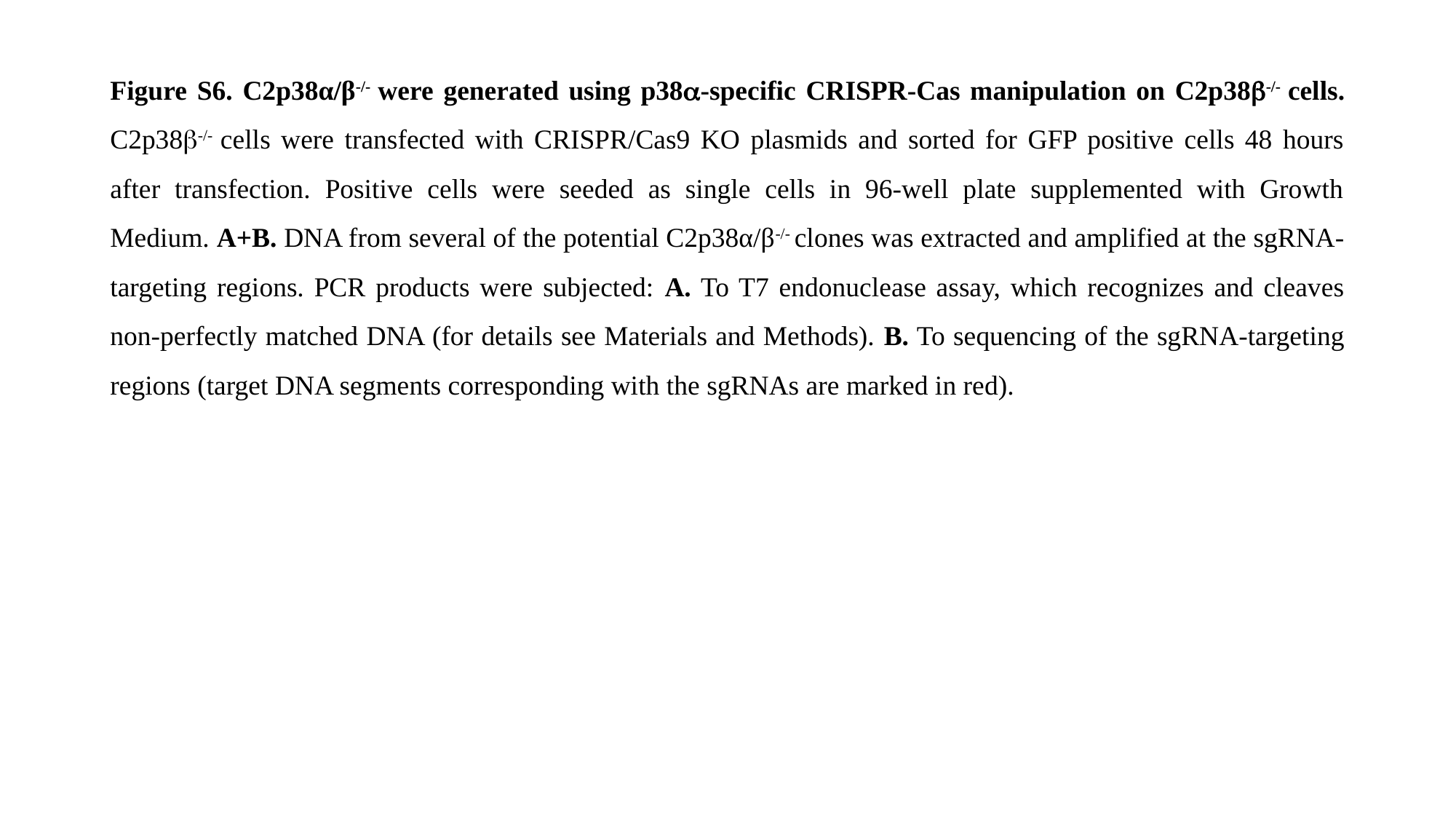

Figure S6. C2p38α/β-/- were generated using p38a-specific CRISPR-Cas manipulation on C2p38b-/- cells. C2p38b-/- cells were transfected with CRISPR/Cas9 KO plasmids and sorted for GFP positive cells 48 hours after transfection. Positive cells were seeded as single cells in 96-well plate supplemented with Growth Medium. A+B. DNA from several of the potential C2p38α/β-/- clones was extracted and amplified at the sgRNA-targeting regions. PCR products were subjected: A. To T7 endonuclease assay, which recognizes and cleaves non-perfectly matched DNA (for details see Materials and Methods). B. To sequencing of the sgRNA-targeting regions (target DNA segments corresponding with the sgRNAs are marked in red).

## Slide 19
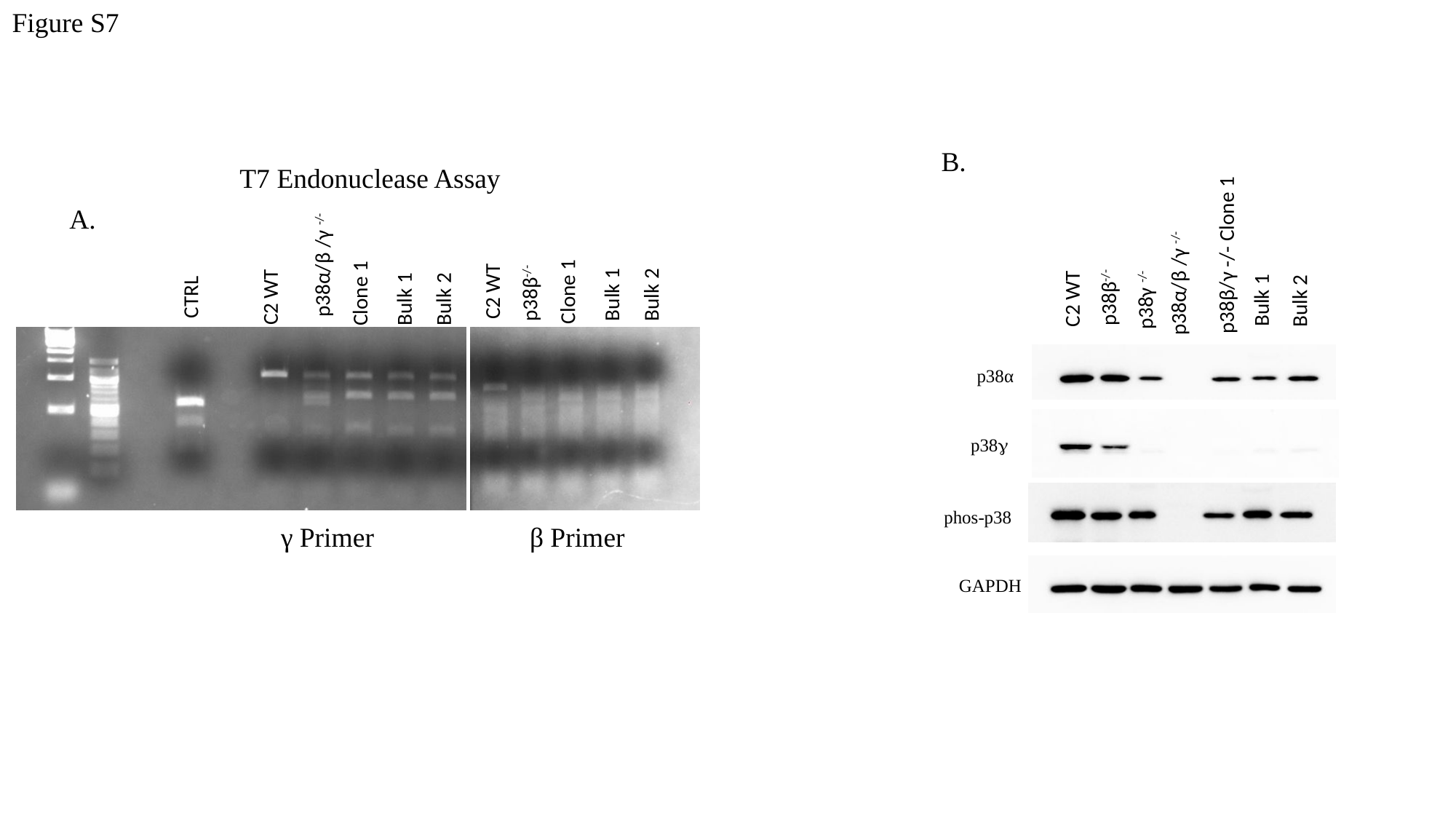

Figure S7
B.
T7 Endonuclease Assay
A.
p38α/β /γ -/-
p38β/γ -/- Clone 1
p38γ -/-
p38α/β /γ -/-
Bulk 1
Bulk 2
Clone 1
Clone 1
Bulk 1
Bulk 2
Bulk 1
Bulk 2
C2 WT
p38β-/-
C2 WT
CTRL
p38β-/-
C2 WT
p38α
p38g
 phos-p38
γ Primer
β Primer
GAPDH

## Slide 20
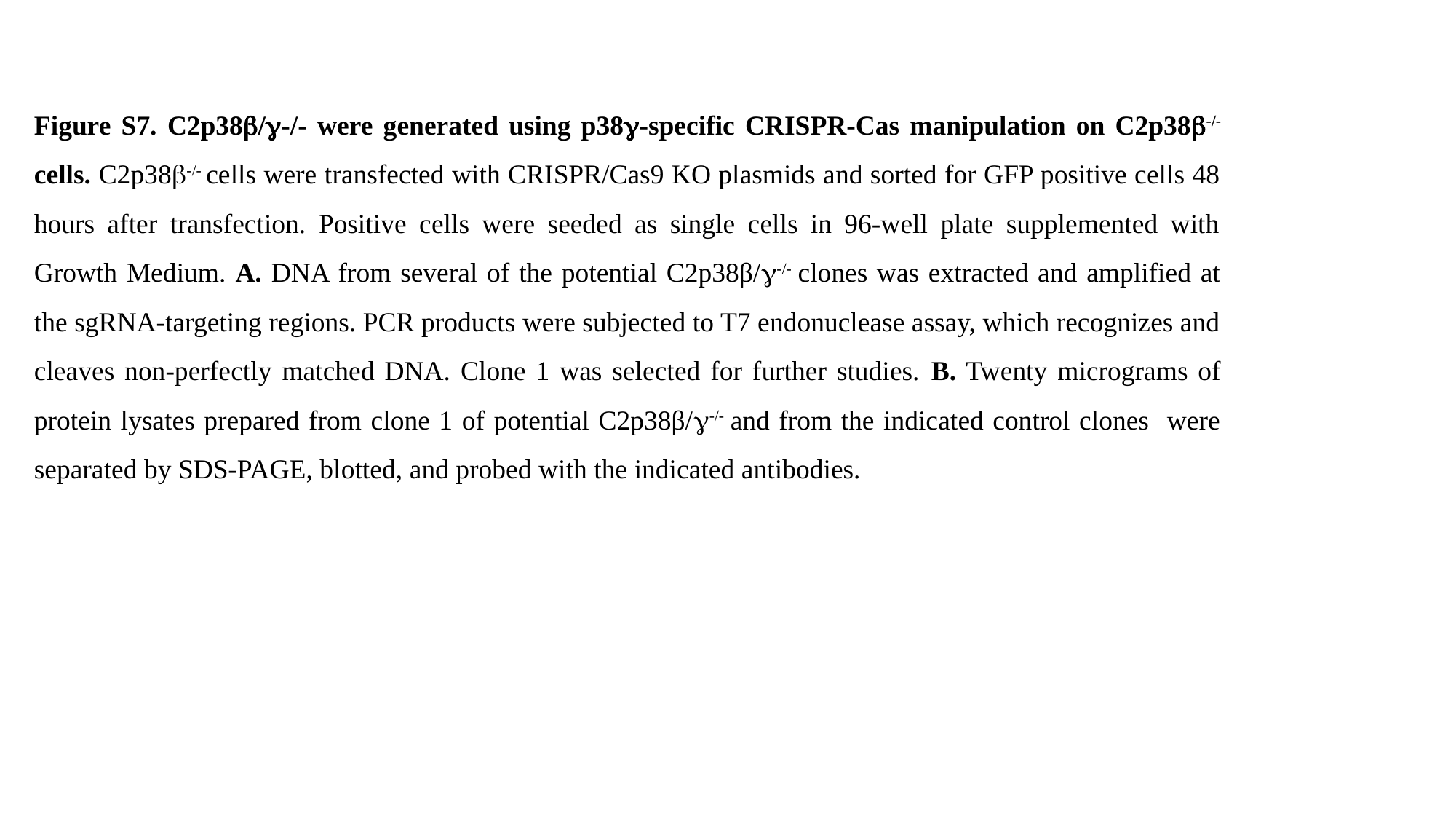

Figure S7. C2p38b/g-/- were generated using p38g-specific CRISPR-Cas manipulation on C2p38b-/- cells. C2p38b-/- cells were transfected with CRISPR/Cas9 KO plasmids and sorted for GFP positive cells 48 hours after transfection. Positive cells were seeded as single cells in 96-well plate supplemented with Growth Medium. A. DNA from several of the potential C2p38β/g-/- clones was extracted and amplified at the sgRNA-targeting regions. PCR products were subjected to T7 endonuclease assay, which recognizes and cleaves non-perfectly matched DNA. Clone 1 was selected for further studies. B. Twenty micrograms of protein lysates prepared from clone 1 of potential C2p38β/g-/- and from the indicated control clones were separated by SDS-PAGE, blotted, and probed with the indicated antibodies.

## Slide 21
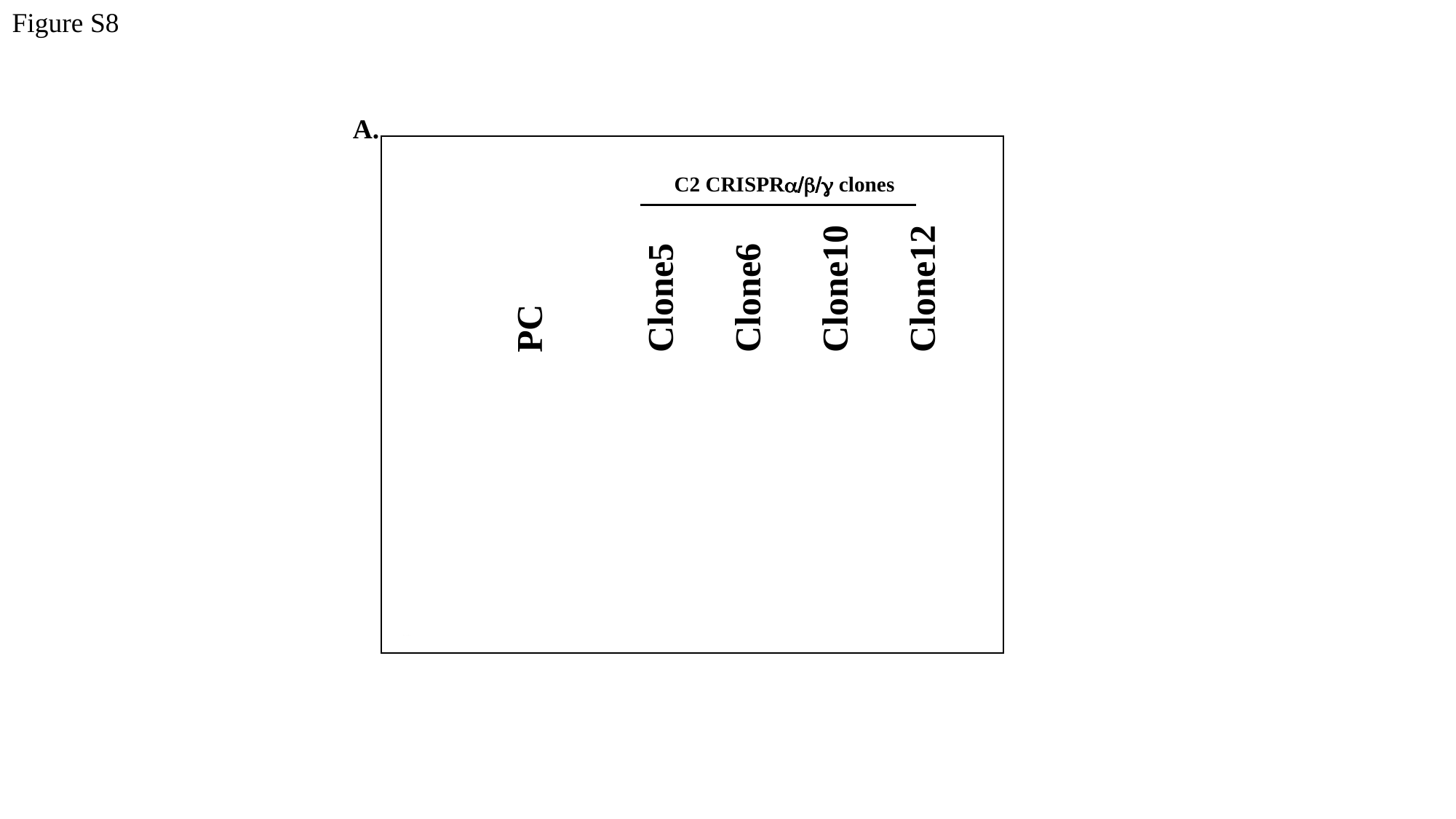

Figure S8
PC
Clone5
Clone6
Clone10
Clone12
A.
C2 CRISPRa/b/g clones

## Slide 22
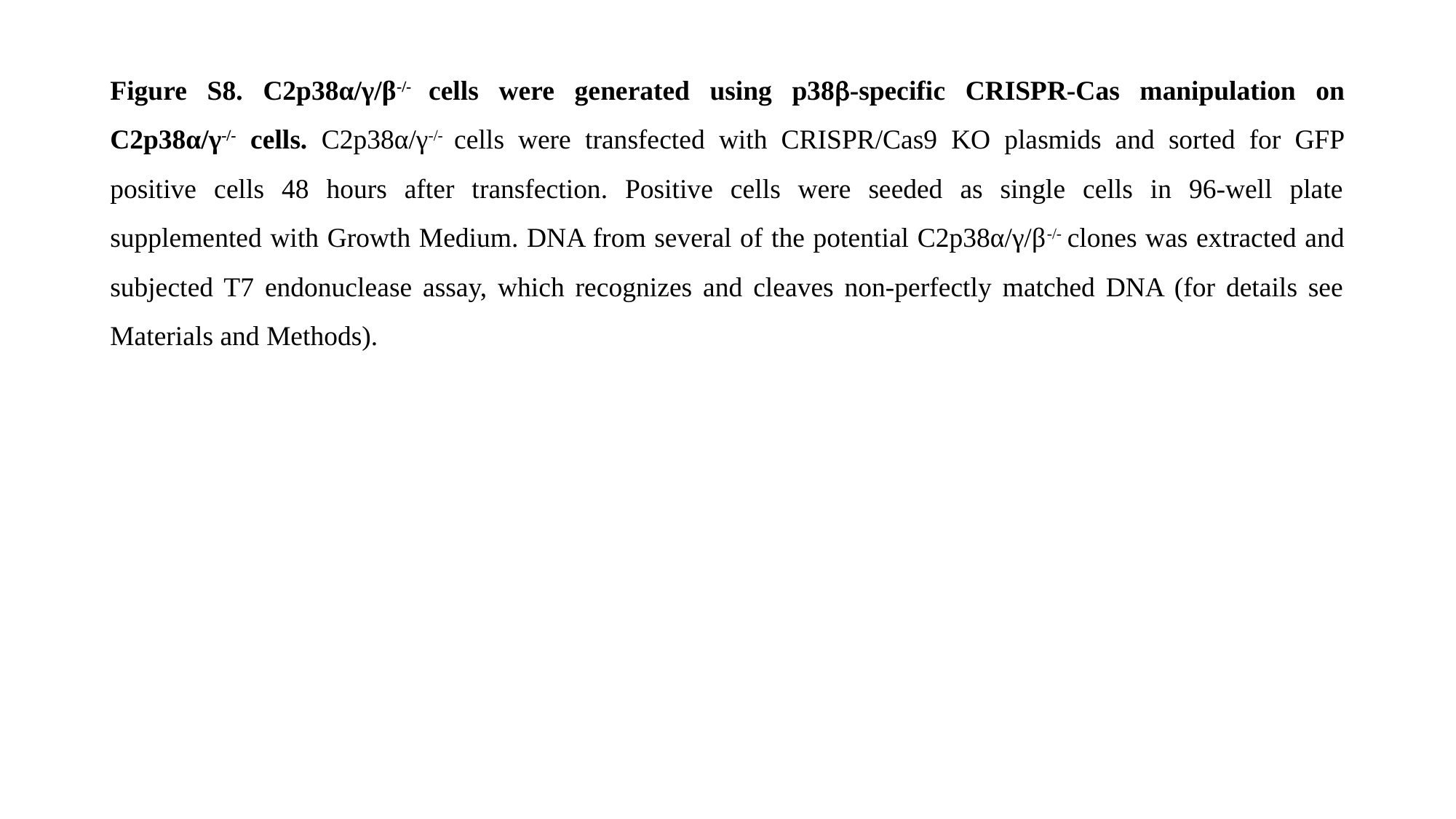

Figure S8. C2p38α/γ/β-/- cells were generated using p38b-specific CRISPR-Cas manipulation on C2p38α/γ-/- cells. C2p38α/γ-/- cells were transfected with CRISPR/Cas9 KO plasmids and sorted for GFP positive cells 48 hours after transfection. Positive cells were seeded as single cells in 96-well plate supplemented with Growth Medium. DNA from several of the potential C2p38α/γ/β-/- clones was extracted and subjected T7 endonuclease assay, which recognizes and cleaves non-perfectly matched DNA (for details see Materials and Methods).

## Slide 23
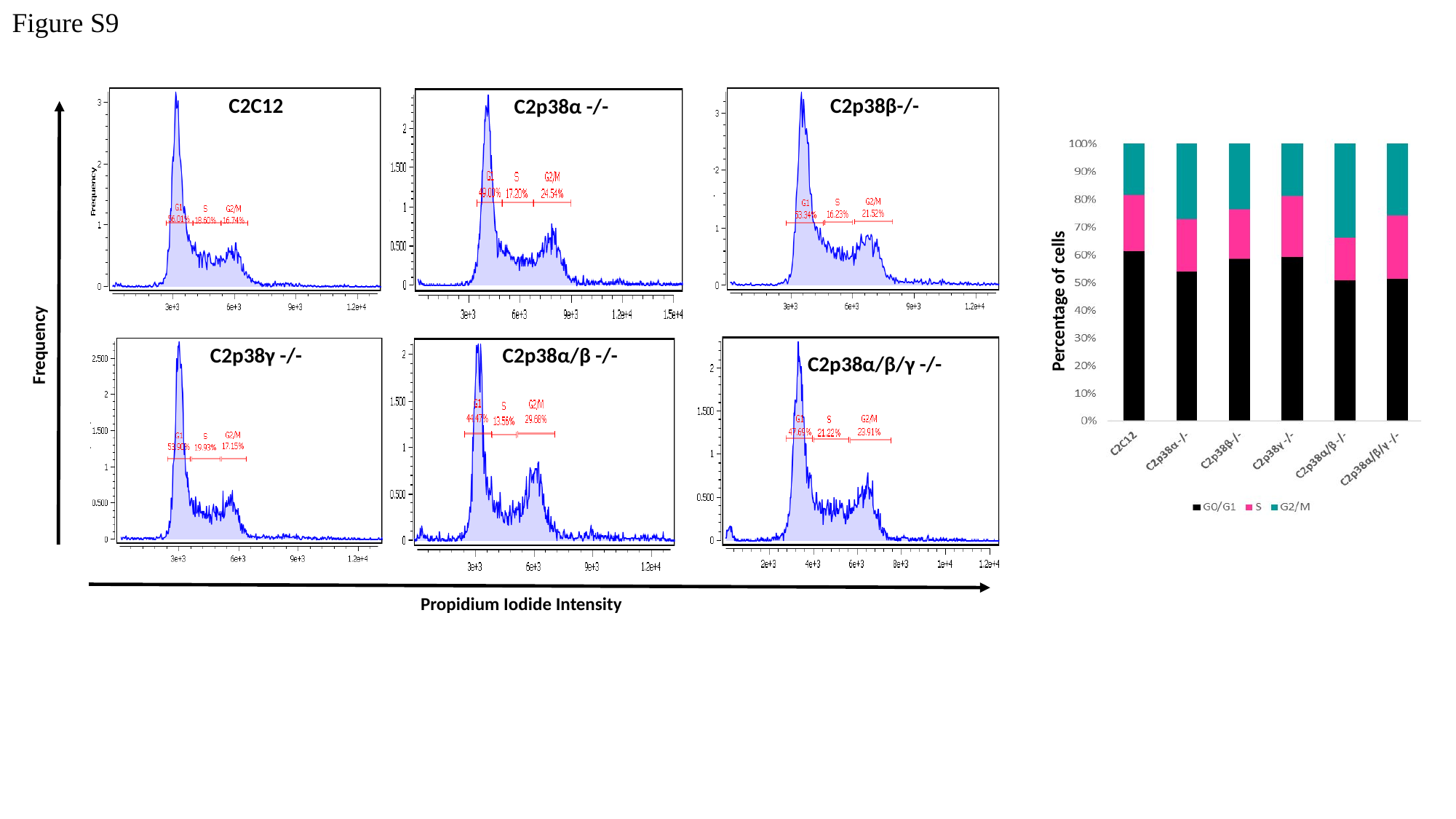

Figure S9
C2p38β-/-
C2C12
C2p38α -/-
Frequency
C2p38γ -/-
C2p38α/β -/-
C2p38α/β/γ -/-
Propidium Iodide Intensity
Percentage of cells

## Slide 24
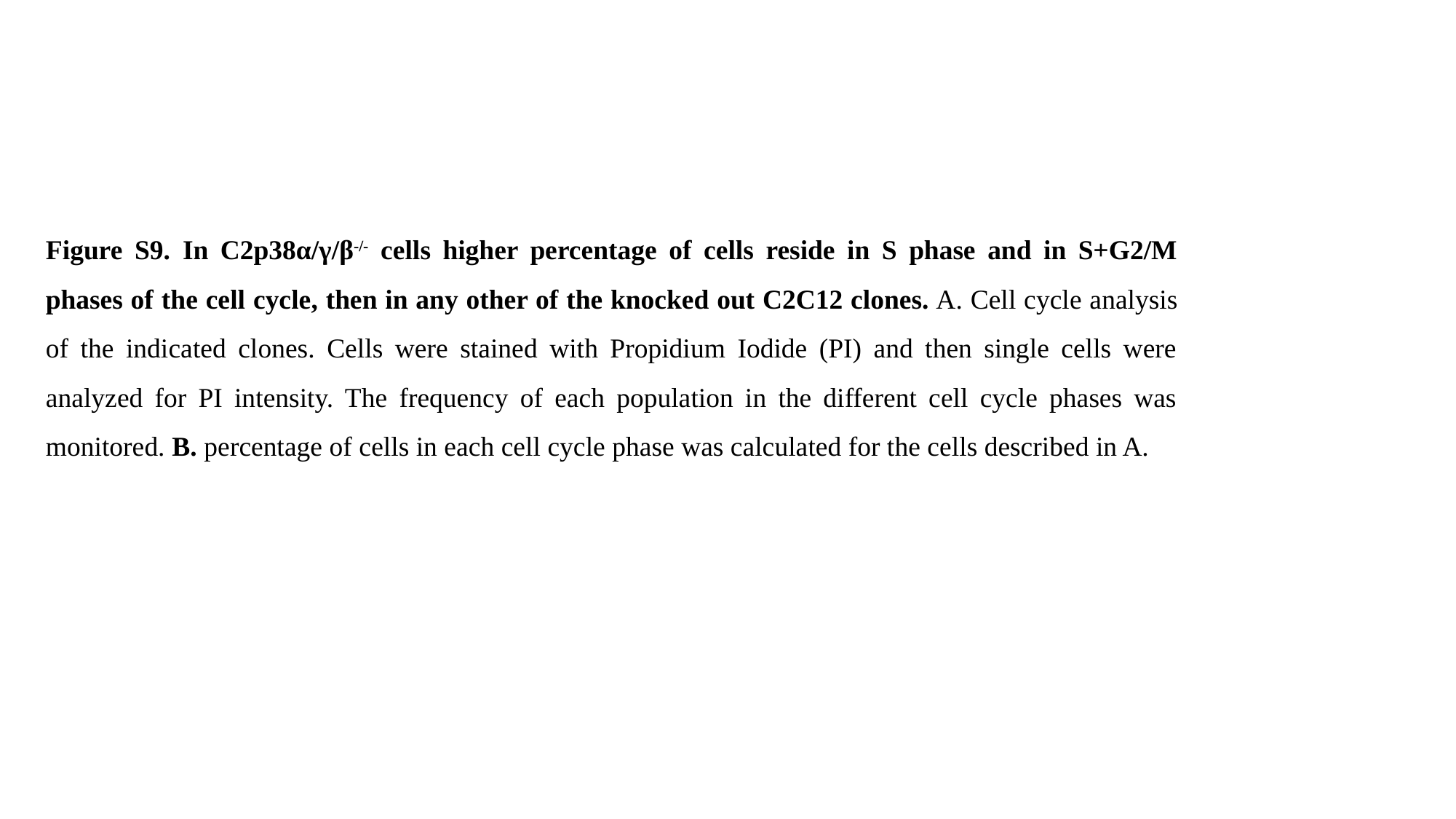

Figure S9. In C2p38α/γ/β-/- cells higher percentage of cells reside in S phase and in S+G2/M phases of the cell cycle, then in any other of the knocked out C2C12 clones. A. Cell cycle analysis of the indicated clones. Cells were stained with Propidium Iodide (PI) and then single cells were analyzed for PI intensity. The frequency of each population in the different cell cycle phases was monitored. B. percentage of cells in each cell cycle phase was calculated for the cells described in A.
